# [PRION^+^] States Are Associated with Specific Histone H3 Post-Translational Modification Changes

**DOI:** 10.3390/pathogens11121436

**Published:** 2022-11-29

**Authors:** Samantha N. Cobos, Chaim Janani, Gabriel Cruz, Navin Rana, Elizaveta Son, Rania Frederic, Jailene Paredes Casado, Maliha Khan, Seth A. Bennett, Mariana P. Torrente

**Affiliations:** 1 Ph.D. Program in Chemistry, The Graduate Center of the City University of New York, New York, NY 10016, USA; 2 Chemistry Department, Brooklyn College, Brooklyn, NY 11210, USA; 3 Biology Department, Brooklyn College, Brooklyn, NY 11210, USA; 4 Graduate Program in Biochemistry, The Graduate Center of the City University of New York, New York, NY 10016, USA; 5 Ph.D. Programs in Chemistry, Biochemistry, and Biology, The Graduate Center of the City University of New York, New York, NY 10016, USA

**Keywords:** *Saccharomyces cerevisiae*, yeast, prion, protein aggregates, histone post-translational modifications, epigenetics

## Abstract

Prions are proteins able to take on alternative conformations and propagate them in a self-templating process. In *Saccharomyces cerevisiae*, prions enable heritable responses to environmental conditions through bet-hedging mechanisms. Hence, [PRION^+^] states may serve as an atypical form of epigenetic control, producing heritable phenotypic change via protein folding. However, the connections between prion states and the epigenome remain unknown. Do [PRION^+^] states link to canonical epigenetic channels, such as histone post-translational modifications? Here, we map out the histone H3 modification landscape in the context of the [SWI^+^] and [PIN^+^] prion states. [SWI^+^] is propagated by Swi1, a subunit of the SWI/SNF chromatin remodeling complex, while [PIN^+^] is propagated by Rnq1, a protein of unknown function. We find [SWI^+^] yeast display decreases in the levels of H3K36me2 and H3K56ac compared to [swi^−^] yeast. In contrast, decreases in H3K4me3, H3K36me2, H3K36me3 and H3K79me3 are connected to the [PIN^+^] state. Curing of the prion state by treatment with guanidine hydrochloride restored histone PTM to [prion^−^] state levels. We find histone PTMs in the [PRION^+^] state do not match those in loss-of-function models. Our findings shed light into the link between prion states and histone modifications, revealing novel insight into prion function in yeast.

## 1. Introduction

Prions, proteins with the ability to adopt self-propagating conformations, have been found throughout the eukaryotic proteome [1]. These infectious agents play a role in the development of transmissible encephalopathies, such as kuru in humans, mad cow disease in cows, and scrapie in sheep [2]. Due to this association, prions have been assumed to play an obstructive role in cellular biology. However, in the baker’s yeast *Saccharomyces cerevisiae*, prions may enable advantageous responses to environmental stress [3,4,5,6].

Over 20 unique prion proteins have been discovered in *S. cerevisiae.* Yeast prions can lead to positive cellular outcomes, such as improved cellular fitness and thicker cell walls [7,8]. Some yeast prion proteins include Sup35, Rnq1, Swi1, and Snt1, responsible for propagating the [PSI^+^], [PIN^+^], [SWI^+^], and [ESI^+^] prion states, respectively [9,10,11,12,13].

Swi1 is a component of the SWI/SNF chromatin remodeling complex. Swi1 is able to bind DNA directly [14]. [SWI^+^], the prion state propagated by Swi1, abolishes flocculin gene expression [15]. Flocculins are proteins responsible for cell–cell and cell-surface adhesion, which allow for yeast to maintain facultative multicellularity [16]. Yeast multicellularity includes pseudohyphal growth of diploid cells [16]. The [SWI^+^] state results in a complete loss of pseudohyphal growth [15]. Yeast can control pseudohyphal growth depending on environmental conditions, and the presence of [SWI^+^] is hypothesized to be a major player in this switch [15].

Rnq1 (Rich in Asparagine (N) and Glutamine (Q) 1) is prion protein responsible for the [PIN^+^] prion state [12]. Interestingly, Rnq1′s function in the non-prion state has yet to be determined. [PIN^+^] has been found to enhance the de novo formation of [PSI^+^], another yeast prion [12]. Null mutants show no phenotype [17]; however, its coexistence with other prions have alluded to Rnq1 playing a larger role in controlling overall gene expression [18].

Some yeast prions, such as the [ESI^+^] state propagated by Snt1, have a deeper role in yeast biology [13]. Snt1 is a scaffold of an essential epigenetic modulator, the histone deacetylase (HDAC) Set3C [19]. [ESI^+^] leads to the formation of an activated chromatin state that can be inherited through generations [13]. The identification of Snt1 as a prion suggests that other yeast prions may also interact with the epigenetic landscape.

Epigenetics refers to heritable changes in the phenotype of an organism that occur without changes in the underlying DNA sequence [20]. DNA is organized and compacted into chromatin. Chromatin structure can regulate gene expression. The basic unit of chromatin, termed a nucleosome, consists of DNA wrapped around a histone octamer core (containing two copies each of histone H2A, H2B, H3 and H4). One key mode of epigenetic regulation includes the post-translational modification (PTM) of histone proteins. The N-terminal tails of histones protrude out of the nucleosome and are modified with a multitude of chemical moieties (including methyl, acetyl, and phosphate groups) on various, but specific residues [20].

Histone PTMs do not only directly impact the binding between DNA and histones, but also serve as binding platforms for other proteins. “Reader” proteins bind PTMs and lead to transcriptional regulation [21]. The location and type of modification determines whether transcription will be activated or silenced. For instance, acetylated lysine residues are “read” by bromodomains, which lead to gene activation [22]. Furthermore, there are histone PTMs “writers” which install these moieties while “erasers” remove them [21]. For example, lysine residues are acetylated by histone acetyltransferases (HATs) and deacetylated by histone deacetylases (HDACs) [23,24].

Prion states are able to elicit phenotypic changes without changing an organism’s DNA sequence. As such, prions have been proposed as an alternative epigenetic mechanism [2]. Moreover, the presence of prions plays a broad role in gene expression [25,26]. These findings hint at a link between prions and other forms of epigenetic modification. Despite this, a comprehensive study of the interplay between yeast prions and histone modifications has not been carried out to date. Here, we establish an association between [PRION^+^] states and histone modifications, by showing that [SWI^+^] and [PIN^+^] each connect to distinct histone H3 PTM patterns. In particular, we find that [SWI^+^] is associated with decreases in the levels of H3K36me2 and H3K56ac compared to [swi^−^] yeast. On the other hand, decreases in H3K4me3, H3K36me2, H3K36me3 and H3K79me3 are linked to the [PIN^+^] state. Furthermore, curing of the [PRION^+^] state restored histone PTM levels to those found in the [prion^−^] state. These changes are distinct to those present in prion protein loss-of-function models. Our findings establish links between prion states and specific histone modifications. While the precise molecular mechanisms by which prions connect to the epigenome remain to be elucidated, establishing an interplay between [PRION^+^] states and the epigenetic landscape expands our knowledge of prion function.

## 2. Results

### 2.1. Different [PRION^+^] States Connect to Unique Histone PTM Alterations

To determine the impact of [PRION^+^] states on the yeast epigenome, we characterized the levels of histone H3 PTMs in [SWI^+^] and [PIN^+^] yeast. Given Swi1′s involvement in chromatin remodeling, we were particularly interested in the [SWI^+^] state and its interplay with epigenetic mechanisms. In fact, the SWI/SNF complex has been previously implicated in transcriptional memory [27]. Similarly, we were interested in [PIN^+^] as Rnq1 is a prion protein whose function is unknown. As such, we posit that probing the connections to the histone PTM landscape in [PIN^+^] yeast may reveal more information about Rnq1′s role.

**Figure 1:**
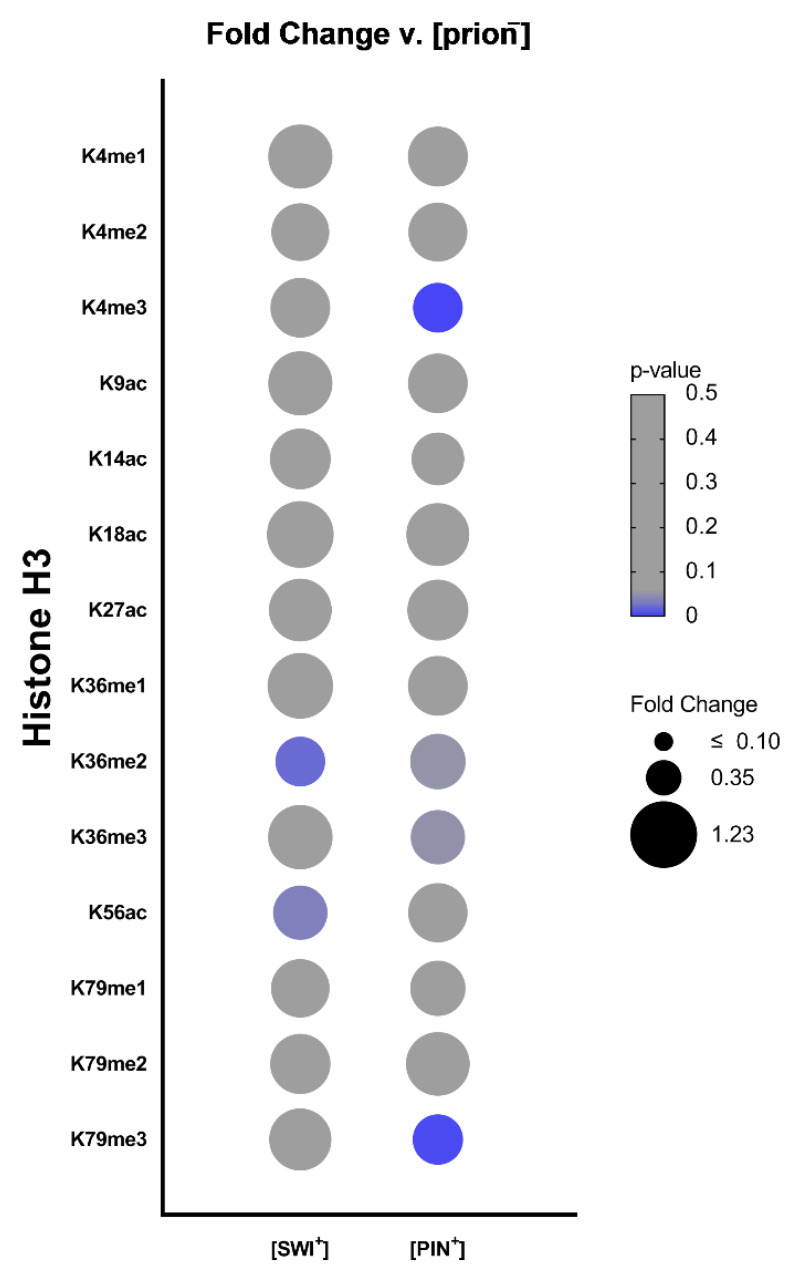
Prion states are connected to distinct changes in the levels of histone H3 post-translational modifications. Bubble plot displaying relative fold change values of histone H3 PTMs in yeast bearing [SWI^+^] and [PIN^+^] compared to [swi^−^] and [pin^−^] cells, respectively. Size of bubbles represents relative fold changes in histone PTM levels. *p* values were calculated using a two-tailed *t* test with Welch’s modification. Color scale represents *p* values; gray indicates a *p* > 0.05, while blue indicates a *p* value ≤ 0.05.

We harvested and lysed both [prion^−^] and [PRION^+^] yeast and separated histone protein from others by sodium dodecyl sulfate-polyacrylamide gel electrophoresis (SDS-PAGE). Individual histone modifications were probed by immunoblotting using commercially available modification-specific antibodies. We focused exclusively on histone H3, as this histone is the most pervasively modified [28]. Altogether, we surveyed 14 histone H3 modifications. Individual PTM levels were quantitated by blot image analysis, normalized to a loading control, and compared with the [prion^−^] state to obtain fold change measurements. Remarkably, we identified distinct histone H3 PTM landscapes for each prion state (Figure 1). Our findings suggest that epigenetic changes are specific to each individual [PRION^+^] and not resulting from non-specific protein aggregation mechanisms. Moreover, we find that [PRION^+^] are connected to global changes in histone PTMs that are apparent without enrichment for specific genomic regions.

### 2.2. [SWI^+^] Is Linked to Decreases in H3K36me2 and H3K56ac Levels

**Figure 2:**
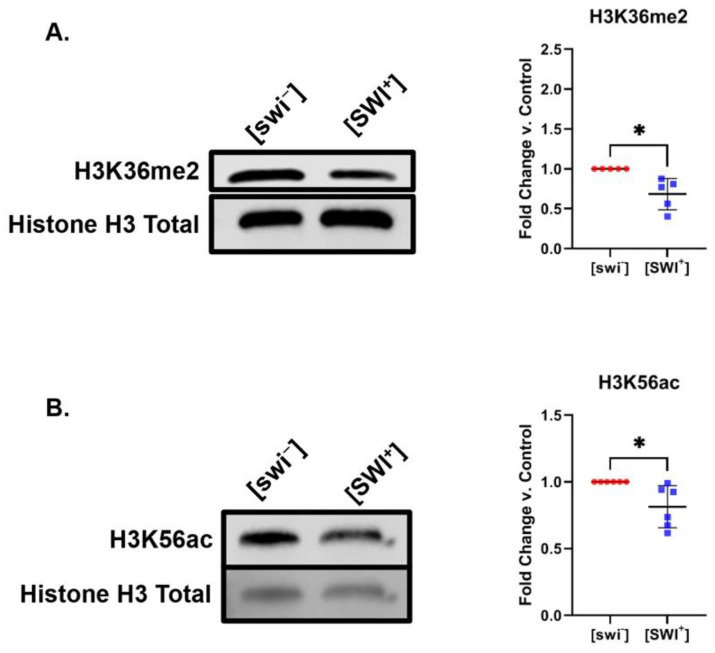
[SWI^+^] is connected to decreases in the levels of H3K36me2 and H3K56ac. Representative immunoblots displaying the levels of (**A**) H3K36me2 and (**B**) H3K56ac for [SWI^+^] and [swi^−^] yeast. Graphs compiling quantification of biological replicates are presented alongside blots. Graphs display the mean fold change in modification levels for each group based on densitometric analysis of Western blots. Error bars indicate the ±SD. *n* = 6 for each experiment, * = *p* < 0.05.

We find [SWI^+^] is connected to modest, but reproducible decreases in the levels of specific PTMs on histone H3. We observe a roughly 30% decrease in levels of H3K36me2 in [SWI^+^] yeast (Figure 2A). H3K36me2 is associated with both gene activation and gene silencing [29] as well as DNA repair [30]. This modification also serves as a binding platform for a number of protein partners [31,32]. For instance, the DNA methyltransferase DNMT3a and the yeast histone deacetylase complex Rpd3 bind H3K36me2 [31,32].

H3K36 methylation is modulated by the histone methyltransferase Set2 in yeast [33]; however, we find no changes in the levels of H3K36me1 and H3K36me3 in [SWI^+^] yeast (Figure S1B). Hence, our data suggests that H3K36me2 alterations do not occur through disruption of Set2 function. HDAC depletion has been found to lead to increases in H3K36me2 specifically (not H3K36me1 or H3K36me3) in fission yeast [34]. Thus, it is possible that disruptions in other histone modifiers can be impacting H3K36me2 alone. Alternatively, crosstalk with other histone modifications could be selectively impacting H3K36me2 over other methylation degrees on this site. Levels of other mono-/di-/and trimethylated lysine residues on histone H3 (K4, K9, K36, and K79) were unchanged in [SWI^+^] yeast (Figure 1 and Figure S1).

[SWI^+^] yeast also displayed a small, but highly reproducible 20% decrease in levels of H3K56ac (Figure 2B). As H3K36me2, H3K56ac plays a role in DNA double-strand break repair [35]. This mark is modulated by the HATs Rtt109 [36] and Gcn5 [35], as well as the HDACs Hst3 and Sir2 [37]. H3K56ac is also an important marker of new nucleosome formation during DNA replication [38]. Notably, this finding was specific to H3K56ac. We did not observe broad changes on histone H3 acetylation. Levels of acetylation on lysines 9, 14, 18, and 27 on histone H3 remained constant in [SWI^+^] yeast compared to [swi^−^] yeast (Figure 1 and Figure S2). We verified linearity of antibody response for selected histone marks (Figure S3). We also corroborated antibody specificity by exploiting yeast strains bearing histone modifying enzyme deletions (Figure S4). Deletion strains also serve as a positive control illustrating the potential range of changes in histone modification levels. We find deletion of Set2 leads to an 80% decrease in levels of H3K36me2 (Figure S4C), while deletion of Rtt109 leads to a 70% decrease in levels of H3K56ac (Figure S4D). Lastly, to ensure that [PIN^+^] was not present in [swi^−^] and [SWI^+^] strains, we probed for Rnq1 aggregates in these cells. We found no Rnq1 aggregates in [swi^−^] and [SWI^+^] strains (Figure S5).

**Figure 3:**
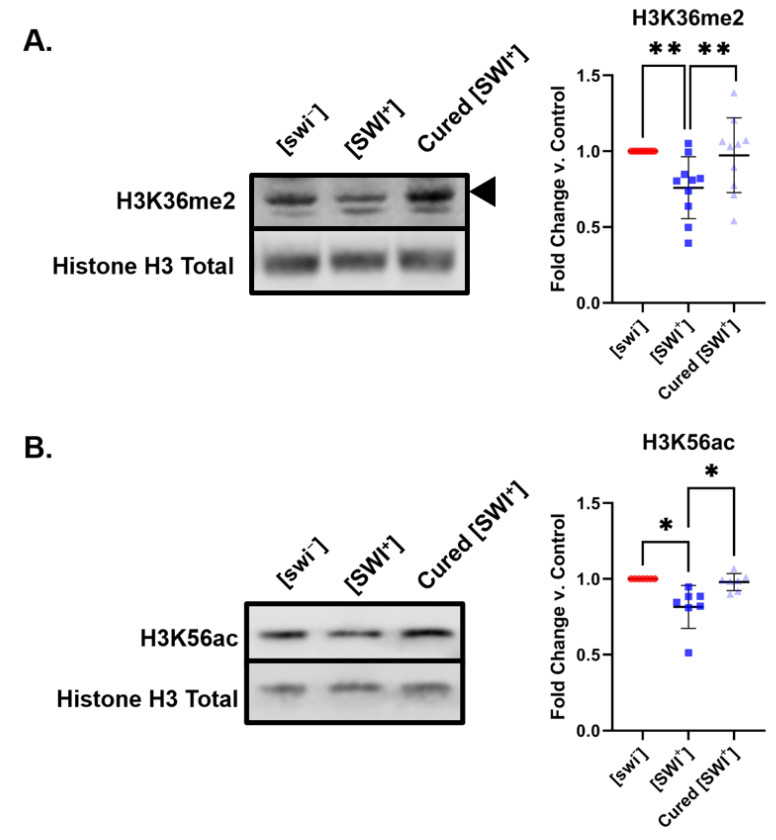
GuHCl curing of [SWI^+^] restores K36me2 and K56ac levels. Representative blots show the levels of (**A**) H3K36me2 (*n* = 10) and (**B**) H3K56ac (*n* = 6) in [swi^−^], [SWI^+^], and cured [SWI^+^] yeast. Graphs compiling quantification of biological replicates are presented alongside blots. Graphs display the mean fold change in modification levels for each group based on densitometric analysis of Western blots. Error bars indicate the ±SD. * = *p* < 0.05, ** = *p* < 0.01.

Upon treatment with GuHCl, yeast can be “cured” of the prion state by inactivation of Hsp104 [39]. Hsp104 is a protein disaggregase native to yeast. Proteins in the [PRION^+^] state form amyloid fibrils. Hsp104 then breaks these fibrils into smaller prion fragments, or “seeds” [40], which are transmitted from mother to daughter cells through the cytoplasm. Once in the daughter cells, these seeds will continue to grow into larger fibrils, repeating the cycle [41]. Inactivation of Hsp104 through GuHCl treatment halts the formation of prion seeds, and effectively cures the yeast of the prion state [42]. Here, we treated [SWI^+^] yeast with 1mM GuHCl. We verified curing of [SWI^+^] by way of cell adhesion tests to show that Swi1 loses its ability to flocculate. After treatment with 1mM GuHCl, we find that cells no longer adhere to each other (Figure S6A). As expected, cells properly disperse into the liquid media and no longer form insoluble pellets. Remarkably, curing [SWI^+^] also reversed the histone PTM disturbances observed in [SWI^+^] yeast. Upon GuHCl treatment, cured [SWI^+^] display H3K36me2 and H3K56ac levels comparable to those in [swi^−^] (Figure 3). This suggests that the changes in H3K36me2 and H3K56ac are indeed linked to the [SWI^+^] prion state in yeast.

### 2.3. Reduced Swi1 Expression Is Associated with Different Histone PTM Changes That Differ from Those Observed in [SWI^+^]

**Figure 4:**
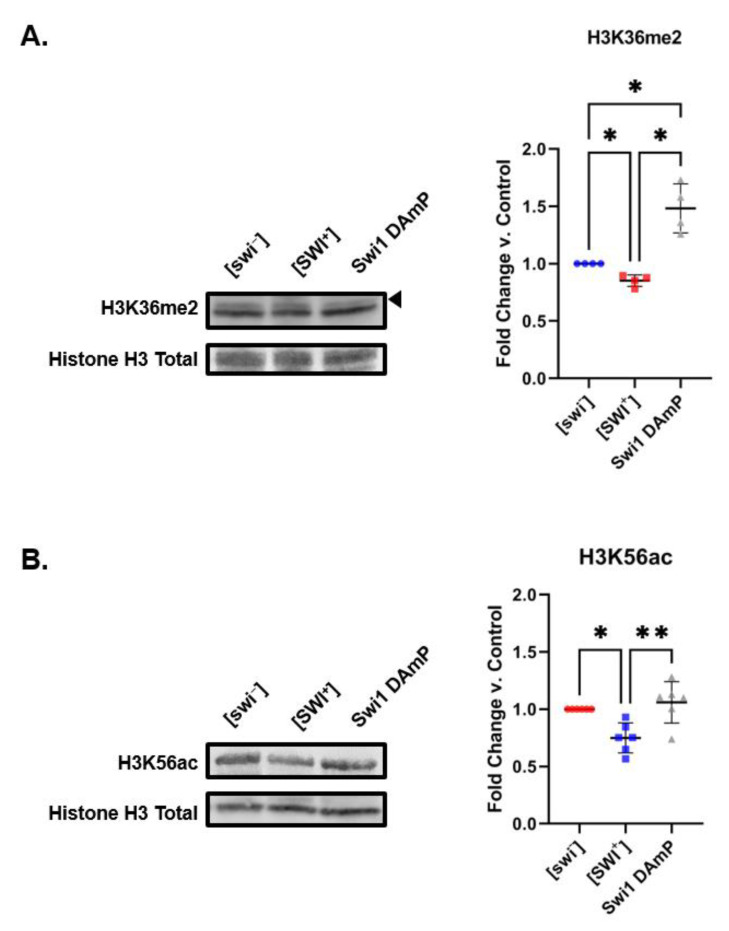
Decreased levels of Swi1 are associated with histone PTM changes that differ from those connected to [SWI^+^]. Representative blots show the levels of (**A**) H3K36me2 and (**B**) H3K56ac [swi^−^], [SWI^+^] and Swi1 DAmP yeast. Graphs compiling quantification of biological replicates are presented alongside blots. Graphs display the mean fold change in modification levels for each group based on densitometric analysis of Western blots. Error bars indicate the ±SD. * = *p* < 0.05, ** = *p* < 0.01.

To determine if the histone PTM changes we describe result from inactivation of the native protein in the [PRION^+^] state or a gain-of-function event, we sought to characterize histone modifications in the context of reduced levels of Swi1. As Swi1 is an essential gene, it cannot be completely knocked out. As an alternative, the Decreased Abundance by mRNA Perturbation (DAmP) Yeast Library is a collection of *S. cerevisiae* strains which gives reduced expression of essential yeast genes to assess their loss of function [43]. Intriguingly, we find that levels of H3K36me2 increase by approximately 50% in Swi1 DAmP yeast compared to both [swi^−^] and [SWI^+^] yeast (Figure 4A). Conversely, H3K56ac levels in Swi1 DAmP yeast were comparable to those found in [swi^−^] (Figure 4B). The lack of overlap between the histone PTM profiles associated with [SWI^+^] and Swi DAmP suggests that [SWI^+^] connects to the epigenome through a mechanism distinct from a mere loss of function. This is in agreement with previous investigations establishing that [SWI^+^] and ΔSwi1 display distinct transcriptomic profiles [25].

### 2.4. [PIN^+^] Is Associated with Decreases in Di- and Trimethylation of Histone H3 at Specific Sites

**Figure 5:**
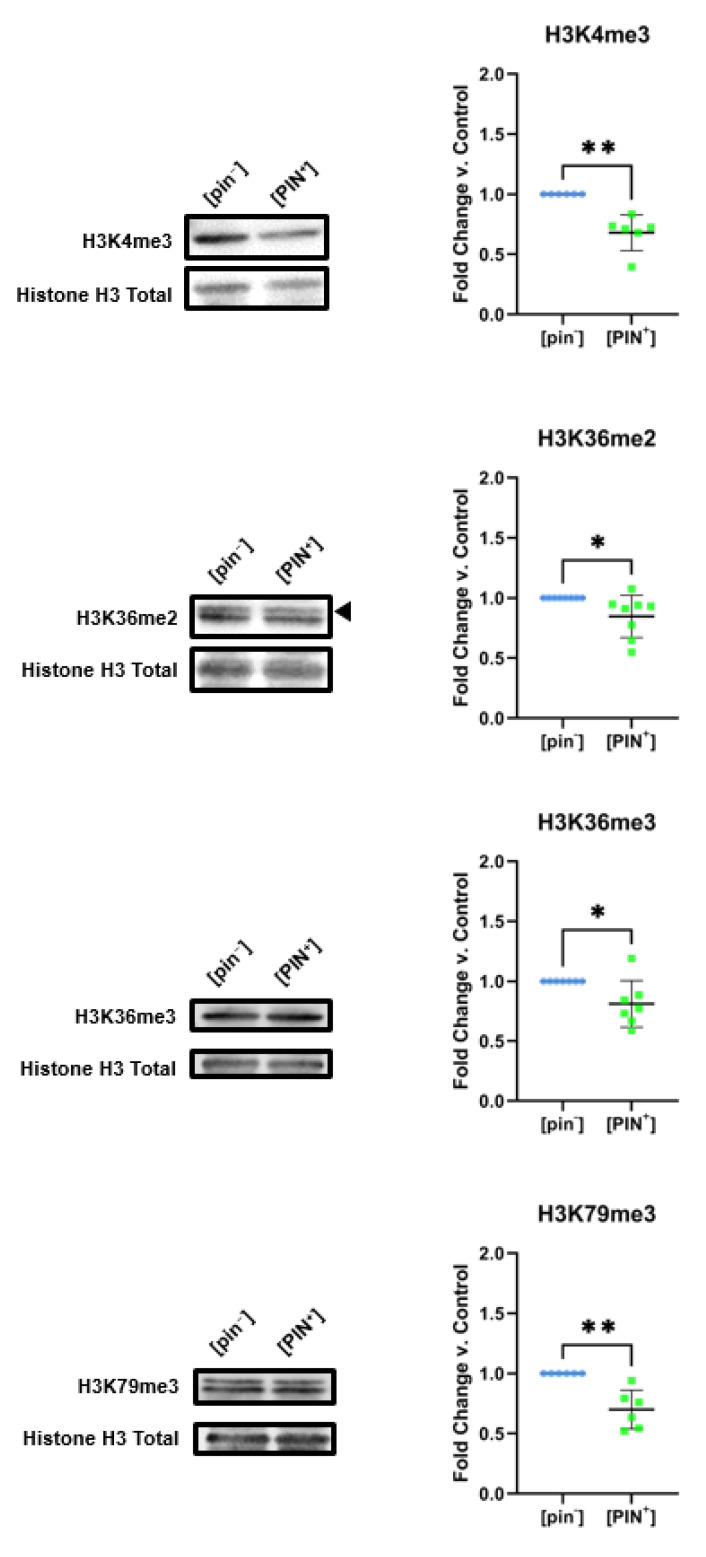
[PIN^+^] is connected to decreases in the di- and trimethylation levels of histone H3 on specific sites. [PIN^+^] is associated with significant decreases in the levels of H3K4me3, H3K36me2, H3K36me3, and H3K79me3 levels compared to [pin^−^] control yeast. Representative immunoblots displaying the levels of each modification for [PIN^+^] and [pin^−^] yeast are shown. Graphs compiling quantification of biological replicates are presented alongside blots. Graphs display the mean fold change in modification levels for each group based on densitometric analysis of Western blots. Error bars indicate the ±SD. *n* = 6 for all experiments, * = *p* < 0.05, ** = *p* < 0.01.

Yeast bearing the [PIN^+^] prion display decreases in specific histone marks when compared to [pin^−^]. Specifically, we find [PIN^+^] is connected to statistically significant decreases in the levels of dimethylation of lysine 36, as well as trimethylation of lysines 4, 36, and 79 on histone H3 (K36me2, H3K4me3, H3K36me3, and H3K79me3, respectively). We detect a roughly 40% decrease in H3K4me3 and H3K79me3 levels in the context of [PIN^+^], while H3K36me2 and H3K36me3 decreased by approximately 30% in [PIN^+^] yeast (Figure 5). Remarkably, we find no changes in the levels of mono- or dimethylation of H3K4 and H3K79 (Figure S7). Hence, it seems decreased di- and tri-methylation on specific sites–rather than global histone hypomethylation–is involved in [PIN^+^] function. We also find that there are no changes in the levels of histone H3 acetylation in [PIN^+^] yeast (Figure S8).

Methylation of H3K4 is controlled by the histone methyltransferase Set1, a subunit of the COMPASS complex [44]. Set1 specifically promotes trimethylation of H3K4 at active gene promoters [45]. As mentioned above, H3K36 methylation is controlled by Set2 [33]. Di- and trimethylation of H3K36 is reduced globally in [PIN^+^] yeast, underscoring a potential role for Set2 in the mechanisms connecting [PIN^+^] to the epigenome. H3K79me3 is installed by the histone methyltransferase Dot1 [46]. Notably, both H3K4me3 and H3K79me3 engage in positive histone crosstalk with H2BK123ub1 [47].

**Figure 6:**
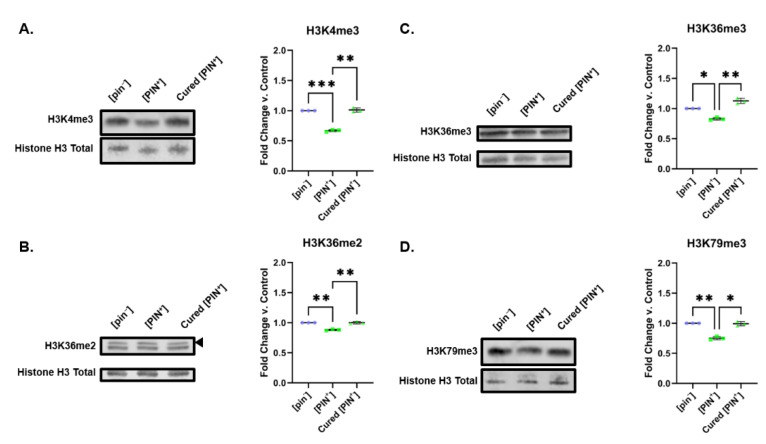
GuHCl treatment of [PIN^+^] yeast restores H3 methylation levels. Representative Western blots showing the levels of (**A**) H3K4me3, (**B**) H3K36me2, (**C**) H3K36me3 and (**D**) H3K79me3 in [pin^−^], [PIN^+^], and cured [PIN^+^] yeast are shown. Graphs compiling quantification of biological replicates are presented alongside blots. Graphs display the mean fold change in modification levels for each group based on densitometric analysis of Western blots. Error bars indicate the ±SD. *n* = 6 for each experiment, * = *p* < 0.05, ** = *p* < 0.01, *** = *p* < 0.001.

As for [SWI^+^], we treated [PIN^+^] cells with 1mM GuHCl to cure the prion state. Several methods exist for verifying the presence of [PIN^+^], including probing for Rnq1-GFP aggregates [48,49]. We chose to probe for Rnq1 aggregates directly using filter retention assays [8,50]. As such, we verified curing of [PIN^+^] by way of filter assays to assess Rnq1′s assembly into SDS-insoluble aggregates. After treatment with 1mM GuHCl, we find a decrease in Rnq1 aggregation (Figure S6B). As expected, perturbation of H3K4me3, H3K36me2/3, and H3K79me3 levels was reversed upon curing of the prion state. Treatment with GuHCl restored relevant histone PTM levels to a level similar to those found in [prion^−^] controls (Figure 6). As for [SWI^+^], these results suggest that the changes in PTM marks are linked to the prion state in yeast.

The phenotypic effects of the prion state can vary depending on the specific yeast genotype. For instance, [PSI^+^] (the prion state of Sup35) conferred variable environmental advantages under a range of various chemotolerance tests in different yeast backgrounds [4]. Thus, we pondered whether changes in the histone PTM landscape connected to [PIN^+^] were influenced by differences in genetic backgrounds. We characterized histone modifications in 74D-694 yeast in the [pin^−^] or [PIN^+^] prion states. Remarkably, H3K4me3, H3K36me2/3, and H3K79me3 levels do not change in 74D [PIN^+^] yeast compared to isogenic [pin^−^] controls (Figure S9). Whereas BY4741 [PIN^+^] yeast reveal decreases in methylation levels (Figure 5), 74D [PIN^+^] yeast display increases in the acetylation levels of H3K9, H3K18 and H3K56 (Figure S10A,B). These changes are also reversible upon GuHCl treatment (Figure S10C), further tying histone PTM changes to the prion state. In addition, several other histone H3 methylation and acetylation marks do not change in the context of 74D [PIN^+^] yeast (Figure S11). Overall, we find distinct histone PTM panoramas for [PIN^+^] in different genetic backgrounds and we cannot identify histone PTMs indicative of the [PIN^+^] state irrespective of genetic background. Alternatively, the divergence in the epigenomic landscape could also be indicative of each strain harboring different variants of [PIN^+^], potentially connecting to different histone PTM landscapes [51]. Further studies on a broader selection of genotypes are required to definitively establish whether these findings are a result of genetic background vs. divergent [PIN^+^] variants.

### 2.5. Rnq1 Deletion Reveals Histone PTM Changes That Differ from Those Observed in [PIN^+^]

**Figure 7:**
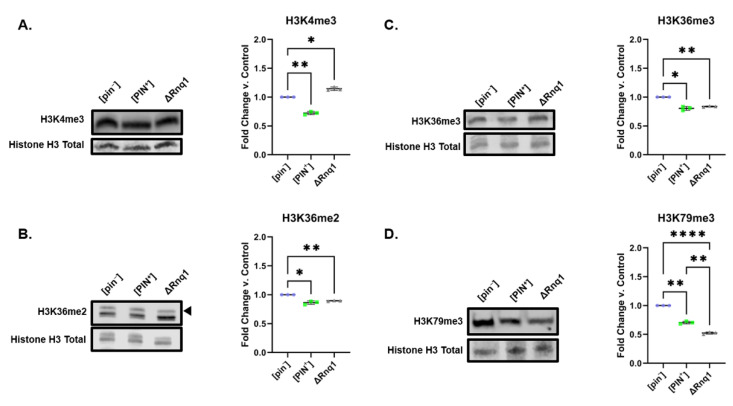
Rnq1 deletion is connected to a different histone PTM landscape from [PIN^+^] yeast. [pin^−^], [PIN^+^] and ΔRnq1 yeast were probed for changes in (**A**) H3K4me3, (**B**) H3K36me2, (**C**) H3K36me3 and (**D**) H3K79me3. Representative blots are shown alongside graphs compiling quantification of biological replicates. Graphs display the mean fold change in modification levels for each group based on densitometric analysis of Western blots. Error bars indicate the ±SD. * = *p* < 0.05, ** = *p* < 0.01, **** = *p* < 0.0001.

As for [SWI^+^] yeast, we were interested in determining if loss of Rnq1 function would link to a histone PTM landscape that was distinct from that of [PIN^+^]. We probed [pin^−^], [PIN^+^], and ΔRnq1 yeast for changes in H3K4me3, H3K36me2, H3K36me3, and H3K79me3 levels (Figure 7). In contrast to [SWI^+^], ΔRnq1 cells overlapped with [PIN^+^] in both direction and magnitude of changes in H3K36me2/3 levels (Figure 7B,C). This suggests Set2 might be impacted by Rnq1’s loss of function. For ease of comparison, we show K36me2 changes side by side for all strains included in this study on a single Western blot (Figure S12). On the other hand–contrary to [PIN^+^]–H3K4me3 levels increased by approximately 20% in ΔRnq1 yeast compared to [pin^−^] yeast (Figure 7A). ΔRnq1 cells show a 50% decrease in H3K79me3 levels when compared to [pin^−^] cells (Figure 7D). This decrease is more robust than that seen in [PIN^+^]. Overall, these results suggest that loss of function mechanisms might underlie [PIN^+^] connection to the epigenome, at least partially.

[PRION^+^] states have been previously linked to large transcriptional changes [25]. Overall, the histone PTM changes we find in [SWI^+^] yeast suggest transcriptional silencing. To further explore this, we chose a straightforward approach in which we measured total RNA level changes from [prion^−^], [PRION^+^], cured [PRION^+^], and loss of function yeast to determine whether the changes in histone PTM levels correlated with overall changes in gene expression (Figure S13). Indeed, [SWI^+^] and Swi DAmP yeast display approximately a 30% decrease in total RNA levels compared to [swi^−^]. (Figure S13A) This is in agreement with previous work in which global levels of gene expression were found to be reduced in both [SWI^+^] and swi1Δ yeast [25]. Upon GuHCl treatment, [SWI^+^] yeast experienced a near full recovery in RNA levels (Figure S13A). For [PIN^+^], the observed histone changes would suggest transcriptional silencing. Puzzlingly, RNA levels are elevated in [PIN^+^] (Figure S13B). This suggests that other uncharacterized histone modifications (on H4 or H2B for instance) or other epigenetic factors outside our study are impacting gene transcription to a greater degree. Nevertheless, upon GuHCl treatment, [PIN^+^] yeast RNA levels to a level comparable to that of [pin^−^] (Figure S13B). ΔRnq1 displays RNA levels lower than [pin^−^] (Figure S13B). The histone PTM alterations we characterize in 74D [PIN^+^] yeast allude to transcriptional activation. Correspondingly, 74D [PIN^+^] yeast display an increase in total RNA levels as compared to 74D [pin^−^] controls (Figure S10E). Upon treatment of 74D [PIN^+^] with GuHCl, RNA levels decrease to 74D [pin^−^] levels (Figure S10E). To exclude the possibility of discrepancy in cell sizes between [prion^−^] and [PRION^+^] strains as well as differential efficiency of RNA extraction from various cell strains, we verified cell morphology and size through microscopy after zymolase treatment (Figure S14). All [SWI^+^] strains were similar to each other, while [PIN^+^] strains in the BY4741 and 74D background were comparable within each set.

## 3. Discussion

**Figure 8:**
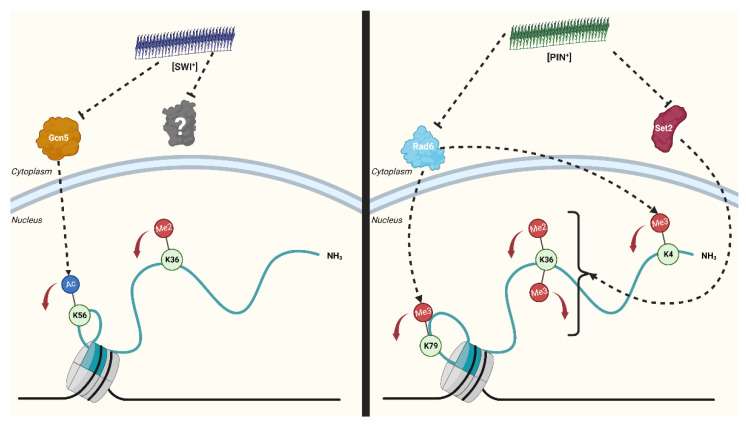
Distinct [PRION^+^] states are connected to unique changes in post-translational modifications on histone H3. [SWI^+^] is linked to decreases in H3K56ac potentially occurring through interference of histone deacetylase Gcn5 (orange) activity. Decreases in H3K36me2 levels could involve other histone modifying enzymes (gray) aside from Set2. [PIN^+^] yeast display a decrease in H3K4me3 and H3K79me3 through potential disruption of the activity of the ubiquitin conjugating enzyme Rad6 (blue). H3K36me2 and H3K36me3 levels reductions in [PIN^+^] yeast could be mediated through potential interactions with the histone methyltransferase Set2 (red). Red arrows represent decreases in associated histone post-translational modification levels. Created with BioRender.com (accessed on 16 November 2022).

Here, we have characterized the epigenome connected to distinct [PRION^+^] states and revealed novel links between two yeast prions and the histone H3 PTM landscape (Figure 8). Surprisingly, we find very little overlap between the histone PTM alterations elicited in each [PRION^+^] state, suggesting these alterations are not linked to general protein aggregation pathways. Both [SWI^+^] and [PIN^+^] are associated with specific alterations in histone H3. These changes are reversible upon curing of the [PRION^+^] phenotype. While it is difficult to definitively establish causal relationships from our experiments, our prion curing results demonstrate a clear link between the prion state and histone PTM alterations. Additionally, total RNA levels were impacted in [PRION^+^] yeast. Disturbances in RNA levels were also reversed by curing of the [PRION^+^] state. It is important to note that we are unable to assign specific biological functions to these histone PTM changes as our experiments characterize genome-wide epigenetic changes and do not pinpoint the specific genes targeted by these alterations.

We find that [SWI^+^] is associated with a decrease in H3K36me2 and H3K56ac, each associated with gene activation [29,52]. Swi1 is a subunit of the SWI/SNF chromatin remodeling complex [14]. Native Swi1 resides mainly in the nucleus [53]; however, in the [SWI^+^] state, Swi1-YFP mutants aggregate in the cytoplasm [54]. The lack of overlap between the histone PTM profiles associated with [SWI^+^] and Swi1 DAmP suggests that [SWI^+^] connects to the epigenome through a mechanism distinct from a mere loss of function. Mislocalization of Swi1 to the cytoplasm in the context of [SWI^+^] may also drag histone modifying enzymes along with it, thus influencing the histone PTM landscape. A hallmark of neurodegenerative disease is the cytoplasmic aggregation of prion-like proteins, which are typically diffuse in the nucleus in their native state [55]. For instance, aggregated FUS mutants associated with amyotrophic lateral sclerosis sequester the histone methyltransferase PRMT1 to the cytoplasm, leading to a decrease in the levels of asymmetric H4R3me2 [56]. Therefore, it is possible that the mislocalization of Swi1 in the [SWI^+^] state also leads to the sequestration of histone modifying enzymes, such as Rtt109 and Gcn5, the histone acetyltransferases which install H3K56ac. Microscopy experiments could illuminate interactions between histone modifying enzymes and [SWI^+^], alas we are unable to expediently carry out such experiments as antibodies for Rtt109 or Gcn5 are not commercially available. Alternatively, it is possible histone PTM disturbances arise from Swi1′s loss of function in [SWI^+^] [11]. While our results suggest a loss-of-function alone is not likely to be at play in the context of [SWI^+^], we cannot exclude a contribution from such a mechanism. Several interactions between mammalian SWI/SNF and histone modifying enzymes have been catalogued, such as the interaction between the Swi1 homologue ARID1A and the histone modifiers HDAC2 and the histone methyltransferase EZH2 [57]. Furthermore, the SWI/SNF-like chromatin remodeler SMARCAD1 associates with various PTM erasers such as HDAC1/2 and the histone methyltransferase G9a/GLP [58]. Hence, it is possible that loss of Swi1 function in [SWI+] disturbs the recruitment of various histone modifying enzymes and at least partially leads to histone PTM alterations.

[PIN^+^] yeast display decreases in H3K4me3, H3K36me2, H3K36me3 and H3K79me3 levels, all marks implicated in gene activation. The reduction of di- and trimethylation of H3K36 in [PIN^+^] yeast underscores a potential role for Set2. Interaction with histone H4 is required for Set2 to install H3K36me2/me3, but it is not required for Set2 to install H3K36me1 [59]. Hence, it is possible that the interaction between Set2 and H4 is disrupted in the context of [PIN+]. Intriguingly, both H3K4me3 and H3K79me3 engage in positive histone crosstalk with H2BK123ub1 [47]. In yeast, H2BK123ub1 is installed by the ubiquitin ligase RAD6/BRE1. Loss of H2BK123ub1 by depletion of RAD6/BRE1 or mutation of the site causes severe loss of H3K4me3 and H3K79me3 [60,61]. Hence, it is possible that disruption of histone H2B ubiquitination is responsible for the selective decrease in H3K4me3 and H3K79me3, without disruption of other degrees of methylation on these sites.

In contrast to [SWI^+^], ΔRnq1 cells overlapped with [PIN^+^] in direction of changes in H3K36me2/3 and H3K79me3. Overall, these results suggest that loss of function mechanisms might underlie [PIN^+^] connection to the epigenome, at least partially. Notably, a different genotype results in a different epigenetic profile for [PIN^+^]. Previous work reveals that [PSI^+^] elicits varied responses to external stresses dependent on yeast genotype [3,4,5,6]. Further investigation by co-immunoprecipitation of histone modifiers could reveal more information about the exact mechanisms linking [PIN^+^] to histone modifiers in different genetic backgrounds, but again, these experiments are complicated by the lack of commercially available antibodies against yeast histone modifiers. Importantly, as [PIN^+^] and ΔRnq1 yeast display different epigenetic landscapes compared to [pin^−^], our data suggests that Rnq1 might have a previously undescribed role in chromatin remodeling.

In both cured [SWI^+^] and cured [PIN^+^] yeast, we find that RNA levels return to those seen in [prion^−^] yeast, suggesting that changes in global gene expression are linked to the prion state. We find that Swi1 DAmP yeast display RNA levels comparable to those observed in [SWI^+^] yeast. In contrast, the effects of [SWI^+^] on gene expression were previosuly found to be weaker than those of Δswi1 when compared to [swi^−^] controls in isogenic 1-4-1-1-D931 strains [62]. As for histone modifications, it is likely genetic background impacts gene expression and RNA levels. In the case of [PIN+], we find that [PIN^+^] and ΔRnq1 RNA levels diverge in both magnitude and direction, suggesting that the changes in overall gene expression in [PIN+] result from some mechanism distinct from Rnq1′s loss of function. Previous work in carried out in the context of Rnq1 overexpression found that several genes were differentially expressed in BY4741 [PIN^+^] and [pin^−^] yeast [63]. Out of the 23 upregulated genes, the majority corresponded to molecular chaperones and stress-related proteins, such as Hsp104, while many of the 27 downregulated genes were involved in cytokinesis, altogether suggesting that Rnq1 overexpression may cause defects in the cell cycle [63]. Further RNA-seq experiments can pinpoint specific genes differentially regulated in [PIN+] and ΔRnq1.

Largely, discussion of prion function invokes loss of function phenotypes of the native protein. For instance, [PSI^+^] results in a loss of Sup35′s translation termination functionality [64]. Our findings highlight histone modifications as conduits for [PRION^+^] states to elicit phenotypic change. Furthermore, the lack of overlap between the histone PTM panoramas connected to [SWI^+^] and [PIN^+^] hint at specificity of biological relevance. Interplay with histone modifications can constitute an alternative, or perhaps complementary mechanism in [PRION^+^] function. On the basis of our results, chromatin immunoprecipitation experiments against H3K36me2 and H3K56ac in [swi^−^]/[SWI^+^] strains and H3K4me3, H3K36me2, H3K36me3 and K3K79me3 in [pin^−^]/[PIN^+^] strains would reveal which genes are impacted by these histone PTM changes and contribute to phenotype alterations. In the case of [PIN^+^], these genes might reveal valuable information about Rnq1′s function. Furthermore, future comprehensive characterization of modifications on other histone proteins–including histone H4 and H2B–will render a more complete picture of the [PRION^+^] epigenome. Overall, our results call attention to other potential mechanisms–separate from loss of function–by which fungal prions elicit phenotypic changes.

## 4. Materials and Methods

### 4.1. Materials

All chemicals are from Sigma-Aldrich (St. Louis, MO, USA) unless otherwise specified.

### 4.2. Yeast Strains

Isogenic S. cerevisiae [SWI^+^] and [swi^−^] strains BY4741 MATα his3Δ1 leu2Δ0 met15Δ0 ura3Δ0 flo8::FLO8::HIS3 [SWI^+^] and MATα his3Δ1 leu2Δ0 met15Δ0 ura3Δ0 flo8::FLO8::HIS3 [swi^−^] were a gift from Prof. Liming Li (Northwestern University) [65]. Isogenic S. cerevisiae [pin^−^] and [PIN^+^] strains BY4741 MATa his3-Δ1 leu2-Δ met15-Δ ura3-Δ [psi^−^][pin^−^] and MATa his3-Δ1 leu2-Δ met15-Δ ura3-Δ [psi^−^][PIN^+^] were a gift from Prof. Susan Liebman (University of Nevada, Reno) [5]. Isogenic S. cerevisiae [pin^−^] and [PIN^+^] strains 74D-694 [MATα, ade1-14(UGA), his3, leu2, trp1, ura3] were a gift from Prof. Jonathan S. Weissman (Massachusetts Institute of Technology) and Prof. James Shorter (University of Pennsylvania) [66]. All yeast strains used in this study and their genotypes are listed in Table S1.

### 4.3. Yeast Culture

Glycerol stocks of [PRION^+^], [prion^−^], BY4741, Swi1 DAmP and deletion yeast strains (Horizon Discovery, Waterbeach UK) were stored at −80 °C. Prior to all experiments, [PRION^+^], [prion^−^] and BY4741 yeast were streaked onto YPD and incubated at 30 °C for 2–3 days. Similarly, Swi1 DAmP and deletion strains were streaked onto YPD + 200 μg/mL G418 and incubated at 30 °C for 2–3 days. Strains were then inoculated into YPD liquid media (or YPD + 200 μg/mL G418 for Swi1 DAmP and deletion strains) and grown to saturation overnight at 30 °C with shaking at 200 rpm. Liquid cultures were then standardized to an OD_600_ of 0.3 and allowed to grow for 5 h at 30 °C at 200 rpm until an OD_600_ of 0.6–0.8 was reached [67]. Cells were then pelleted, flash frozen, and stored at −80 °C.

### 4.4. Prion Curing

GuHCl curing was performed as previously described [39]. Briefly, [PRION^+^] yeast on YPD plates were re-streaked onto YPD plates supplemented with 1 mM GuHCl and incubated at 30 °C overnight. Yeast were then grown in YPD liquid media supplemented with 1mM GuHCl overnight at 30 °C at 200 rpm. Cultures were then standardized to an OD_600_ of 0.3 and allowed to grow for 5 h at 30 °C at 200 rpm until an OD_600_ of 0.6–0.8 was reached. Cells were pelleted, flash frozen, and stored at −80 °C.

### 4.5. RNA Purification and Quantification

Frozen yeast pellets were thawed and cell counts were normalized upon counting with a hemocytometer (Thermo Fisher, Waltham, MA, USA; cat. no. 501311352). Cells were then treated with 100 units of Zymolyase-20T (Nacalai USA, San Diego, CA, USA; cat. no. 07663-91) for 30 min at 30 °C. RNA was isolated using an RNeasy Mini Kit from Qiagen (Germantown, MD, USA; cat. no. 74104) according to the manufacturer’s instructions. Total RNA levels were measured in a Qubit 2.0 Fluorometer (Thermo Fisher Scientific), using a Qubit RNA Broad Range (BR) Assay Kit (Thermo Fisher Scientific, cat. no. Q10210). 2 × 10^7^ cells were resuspended in 200 μL of sterile water. 30 μL of this suspension was then pipetted onto an untreated microscope slide (Corning Inc, Corning, NY, USA; cat # CLS294775 × 25). Cells were photographed with a Leica MC120 HD (Deer Park, IL, USA) camera mounted on a Leica DMi1 microscope at 5× magnification. All experiments were repeated a minimum of three times with independent cell samples.

### 4.6. Western Blotting

Frozen yeast pellets were thawed and processed for Western Blotting as outlined in Bennett et al. [67]. Briefly, cell pellets were treated with 0.2 M NaOH for 10 min on ice, pelleted again, and resuspended in 100 µL of 1× SDS sample buffer. Samples were then boiled at 95 °C for 10 min, followed by separation of cell lysates by SDS-PAGE using a 12% polyacrylamide gel and transfer to a PVDF membrane (EMD Millipore, Taunton, MA, USA). Membranes were blocked using LI-COR blocking buffer (LI-COR Biosciences, Lincoln, NE, USA) for 1 h at room temperature with gentle rocking. Primary antibody incubations were performed at 4 °C overnight with rocking. For histone modification detection, all antibodies were purchased from Abcam (Cambridge, MA, USA) unless otherwise specified: Histone H3 Total (cat. no. ab24834), H3K4me1 (cat. no. ab8895), H3K4me2 (cat. no. ab7766), H3K4me3 (cat. no. ab8580), H3K9ac (cat. no. ab10812), H3K14ac (Millipore, cat. no. 07-353), H3K18ac (cat. no. ab1191), H3K27ac (cat. no. ab45173), H3K36me1 (cat. no. ab9048), H3K36me2 (cat. no. ab9049), H3K36me3 (cat. no. ab9050), H3K56ac (ActiveMotif, Carlsbad, CA, USA; cat. no. 39281), H3K79me1 (Millipore, cat. no. ABE213), H3K79me2 (cat. no. ab3594), H3K79me3 (cat. no. ab2621). Blots were processed using goat anti-mouse and goat anti-rabbit secondary antibodies (LI-COR Biosciences) and imaged on an Odyssey Fc Imaging System (LI-COR Biosciences). All experiments were performed a minimum of three times with independent cell samples.

### 4.7. Filter Retention Assay

Filter retention assays were performed as previously described [68]. Frozen yeast aliquots were resuspended in spheroplasting solution (1.2 M D-sorbitol, 0.5 mM MgCl_2_, 20 mM Tris pH 7.5, 50 mM β-mercaptoethanol, 0.5 mg/mL zymolyase 100T) and incubated for 1 h at 30 °C with agitation. Spheroplasts were collected by centrifugation at 1000× g for 5 min and the supernatant was removed. The pellet was resuspended in lysis buffer (20 mM Tris pH 7.5, 10 mM β-mercaptoethanol, 0.025 U/uL benzonase, 0.5% Triton X-100, 2× HALT protease inhibitor). Samples were vortexed at high speed for 1 min, then incubated for 10 min at room temperature. Cellular debris was sedimented by centrifugation at maximum speed for 2 min, and the supernatant was transferred to fresh tubes for processing. The samples were then mixed with 10% SDS in water to a final concentration of 2% SDS and then incubated for 10 min at room temperature with slight agitation. A Bio-Dot Apparatus (BioRad, Hercules, CA, USA; cat. no. 1706545) was then fitted with a water-soaked GB003 blotting paper (MilliporeSigma, Burlington, MA, USA; cat. no. WHA10427810) and a cellulose acetate membrane (GE Healthcare, Chicago, IL, USA; cat. no. 10404044) soaked in PBS containing 0.1%SDS. Samples were gently pipetted into the wells of the Bio-Dot and then filtered through the vacuum manifold, followed by five washes with PBS containing 0.1% SDS. The membrane was then incubated overnight with anti-Rnq1 C-terminus (Abcam, cat. no. 188279, 1:300 dilution) with agitation at 4 °C, followed by four washes with PBST, and an hour-long incubation with a secondary goat anti-rabbit antibody at room temperature with agitation. After four washes with PBST, the membrane was given a final PBS wash, and then imaged on an Odyssey Fc Imaging System. Experiments were independently repeated three times.

### 4.8. Flocculation Measurements

Flocculation assays were carried out as described [69]. After 2 days of growth in either YPD liquid media or YPD media supplemented with 1mM GuHCl at 300 rpm at 30 °C, the flocculating ability of cells was assessed based on appearance of visible cell aggregates. Saturated cell cultures were vigorously vortexed and photographed. Experiments were independently repeated three times.

### 4.9. Data and Statistical Analysis

Densitometric analysis of Western blots and filter retention assays was performed using Image Studio Software (LI-COR Biosciences). Signals obtained for histone modifications were normalized to their respective histone H3 signals (modification/H3 Total = Relative Density), which were then compared to control sample signal values to calculate fold change values (Relative Density of Sample X/Relative Density of Control = Fold Change). Anti-H3K63me2 (Abcam) produces two bands, top-most band at 17kDa was used for quantifications, per manufacturers’ recommendation. Fold change values were then used for statistical analysis. Filter retention assay results were analyzed in the same manner. RNA concentrations were reported as raw values given by the Qubit in μg/mL. Statistical analysis of data was performed in GraphPad Prism. Significant differences between sample groups were determined using two-tailed Welch’s *t*-test with *p* = 0.05 as the cutoff for significance. Error bars represent the standard deviation (SD) calculated from the fold changes obtained.

## 5. Conclusions

To further our knowledge of prion function, we sought to explore the interplay between prion states and histone post-translational modifications. We have revealed that two yeast prions, [SWI^+^] and [PIN^+^], are connected to distinct genome-wide changes in histone PTM levels. These changes were reversed upon curing of the [PRION^+^] state. Histone PTM changes associated with [PRION+] differ from those associated with loss-of-function scenarios. To the best of our knowledge, this is the first study to establish a relationship between prions and histone PTMs, highlighting the potential spectrum of prions’ biological function. Elucidating the exact mechanisms through which prions connect to specific histone post-translational modifications will further our understanding of how prions give rise to heritable changes in phenotype.

## Data Availability

All data is provided within the text and Supplementary Materials.

## Supplementary Materials

The following supporting information can be downloaded at: https://www.mdpi.com/article/10.3390/pathogens11121436/s1, Table S1. List of yeast strains used in this study.; Figure S1. Methylation levels on lysines 4, 36, and 79 of Histone H3 are stable in connection to [SWI+].; Figure S2. Levels of acetylation on lysines 9, 18, 14, and 27 of Histone H3 are unchanged in connection to [SWI^+^].; Figure S3. Linear range of detection for select antibodies.; Figure S4. Deletion of histone modifying enzymes leads to the loss of specific histone post-translational modifications.; Figure S5. [SWI^+^] yeast do not display Rnq1 aggregates.; Figure S6. Treatment of [PRION^+^] yeast with guanidine hydrochloride abolishes prion phenotypes.; Figure S7. Levels of mono- and dimethylation of lysines 4and 79, as well as mono-methylation on lysine 36 on histone H3 remain unchanged in the context of [PIN^+^].; Figure S8. Levels of acetylation of lysines 9, 14, 18, 27 and 56 on histone H3 remain unchanged in the context of [PIN^+^] yeast.; Figure S9. Histone post-translational modification landscape changes caused by [PRION^+^] diverge in different genetic backgrounds.; Figure S10. Histone post-translational modification landscape changes linked to [PIN^+^] diverge in different genetic backgrounds.; Figure S11. [PIN^+^] does not impact select methylation and acetylation levels of histone H3 in 74D-694 yeast.; Figure S12. Changes in H3K36me2 in various yeast strains.; Figure S13. Total RNA levels in [PRION^+^] yeast differ from those in [prion^−^] yeast.; Figure S14. Cell morphology is similar in [prion^−^], [PRION^+^], cured [PRION^+^], and loss-of-function yeast. References [70,71] are cited in supplementary materials.

## References

[1] Aguzzi A., Polymenidou M. (2004). Mammalian prion biology: One century of evolving concepts. Cell.

[2] Halfmann R., Lindquist S. (2010). Epigenetics in the extreme: Prions and the inheritance of environmentally acquired traits. Science.

[3] Namy O., Galopier A., Martini C., Matsufuji S., Fabret C., Rousset J.P. (2008). Epigenetic control of polyamines by the prion [PSI+]. Nat. Cell Biol..

[4] Joseph S.B., Kirkpatrick M. (2008). Effects of the [PSI+] prion on rates of adaptation in yeast. J. Evol. Biol..

[5] True H.L., Lindquist S.L. (2000). A yeast prion provides a mechanism for genetic variation and phenotypic diversity. Nature.

[6] Eaglestone S.S., Cox B.S., Tuite M.F. (1999). Translation termination efficiency can be regulated in Saccharomyces cerevisiae by environmental stress through a prion-mediated mechanism. EMBO J..

[7] Franzmann T.M., Jahnel M., Pozniakovsky A., Mahamid J., Holehouse A.S., Nuske E., Richter D., Baumeister W., Grill S.W., Pappu R.V. (2018). Phase separation of a yeast prion protein promotes cellular fitness. Science.

[8] Alberti S., Halfmann R., King O., Kapila A., Lindquist S. (2009). A systematic survey identifies prions and illuminates sequence features of prionogenic proteins. Cell.

[9] Wickner R.B., Shewmaker F.P., Bateman D.A., Edskes H.K., Gorkovskiy A., Dayani Y., Bezsonov E.E. (2015). Yeast prions: Structure, biology, and prion-handling systems. Microbiol. Mol. Biol. Rev..

[10] Lindquist S., Krobitsch S., Li L., Sondheimer N. (2001). Investigating protein conformation-based inheritance and disease in yeast. Philos. Trans. R. Soc. Lond. B Biol. Sci..

[11] Goncharoff D.K., Du Z., Li L. (2018). A brief overview of the Swi1 prion-[SWI+]. FEMS Yeast Res..

[12] Sondheimer N., Lindquist S. (2000). Rnq1: An epigenetic modifier of protein function in yeast. Mol. Cell.

[13] Harvey Z.H., Chakravarty A.K., Futia R.A., Jarosz D.F. (2020). A Prion Epigenetic Switch Establishes an Active Chromatin State. Cell.

[14] Sudarsanam P., Winston F. (2000). The Swi/Snf family nucleosome-remodeling complexes and transcriptional control. Trends Genet..

[15] Du Z., Zhang Y., Li L. (2015). The Yeast Prion [SWI(+)] Abolishes Multicellular Growth by Triggering Conformational Changes of Multiple Regulators Required for Flocculin Gene Expression. Cell Rep..

[16] Fisher R.M., Regenberg B. (2019). Multicellular group formation in Saccharomyces cerevisiae. Proc. Biol. Sci..

[17] Stevenson L.F., Kennedy B.K., Harlow E. (2001). A large-scale overexpression screen in Saccharomyces cerevisiae identifies previously uncharacterized cell cycle genes. Proc. Natl. Acad. Sci. USA.

[18] Kurahashi H., Shibata S., Ishiwata M., Nakamura Y. (2009). Selfish prion of Rnq1 mutant in yeast. Genes Cells.

[19] Pijnappel W.W., Schaft D., Roguev A., Shevchenko A., Tekotte H., Wilm M., Rigaut G., Seraphin B., Aasland R., Stewart A.F. (2001). The *S. cerevisiae* SET3 complex includes two histone deacetylases, Hos2 and Hst1, and is a meiotic-specific repressor of the sporulation gene program. Genes Dev..

[20] Gibney E.R., Nolan C.M. (2010). Epigenetics and gene expression. Heredity.

[21] Jenuwein T., Allis C.D. (2001). Translating the histone code. Science.

[22] Ntranos A., Casaccia P. (2016). Bromodomains: Translating the words of lysine acetylation into myelin injury and repair. Neurosci. Lett..

[23] Roth S.Y., Denu J.M., Allis C.D. (2001). Histone acetyltransferases. Annu. Rev. Biochem..

[24] Marks P.A., Miller T., Richon V.M. (2003). Histone deacetylases. Curr. Opin. Pharmacol..

[25] Du Z., Regan J., Bartom E., Wu W.S., Zhang L., Goncharoff D.K., Li L. (2020). Elucidating the regulatory mechanism of Swi1 prion in global transcription and stress responses. Sci. Rep..

[26] Baudin-Baillieu A., Legendre R., Kuchly C., Hatin I., Demais S., Mestdagh C., Gautheret D., Namy O. (2014). Genome-wide translational changes induced by the prion [PSI+]. Cell Rep..

[27] Kundu S., Horn P.J., Peterson C.L. (2007). SWI/SNF is required for transcriptional memory at the yeast GAL gene cluster. Genes Dev..

[28] Bannister A.J., Kouzarides T. (2011). Regulation of chromatin by histone modifications. Cell Res..

[29] Wagner E.J., Carpenter P.B. (2012). Understanding the language of Lys36 methylation at histone H3. Nat. Rev. Mol. Cell Biol..

[30] Fnu S., Williamson E.A., De Haro L.P., Brenneman M., Wray J., Shaheen M., Radhakrishnan K., Lee S.H., Nickoloff J.A., Hromas R. (2011). Methylation of histone H3 lysine 36 enhances DNA repair by nonhomologous end-joining. Proc. Natl. Acad. Sci. USA.

[31] Weinberg D.N., Papillon-Cavanagh S., Chen H., Yue Y., Chen X., Rajagopalan K.N., Horth C., McGuire J.T., Xu X., Nikbakht H. (2019). The histone mark H3K36me2 recruits DNMT3A and shapes the intergenic DNA methylation landscape. Nature.

[32] Li B., Jackson J., Simon M.D., Fleharty B., Gogol M., Seidel C., Workman J.L., Shilatifard A. (2009). Histone H3 lysine 36 dimethylation (H3K36me2) is sufficient to recruit the Rpd3s histone deacetylase complex and to repress spurious transcription. J. Biol. Chem..

[33] Krogan N.J., Kim M., Tong A., Golshani A., Cagney G., Canadien V., Richards D.P., Beattie B.K., Emili A., Boone C. (2003). Methylation of histone H3 by Set2 in Saccharomyces cerevisiae is linked to transcriptional elongation by RNA polymerase II. Mol. Cell Biol..

[34] Abshiru N., Rajan R.E., Verreault A., Thibault P. (2016). Unraveling Site-Specific and Combinatorial Histone Modifications Using High-Resolution Mass Spectrometry in Histone Deacetylase Mutants of Fission Yeast. J. Proteome Res..

[35] Tjeertes J.V., Miller K.M., Jackson S.P. (2009). Screen for DNA-damage-responsive histone modifications identifies H3K9Ac and H3K56Ac in human cells. EMBO J..

[36] Han J., Zhou H., Horazdovsky B., Zhang K., Xu R.M., Zhang Z. (2007). Rtt109 acetylates histone H3 lysine 56 and functions in DNA replication. Science.

[37] Kadyrova L.Y., Mertz T.M., Zhang Y., Northam M.R., Sheng Z., Lobachev K.S., Shcherbakova P.V., Kadyrov F.A. (2013). A reversible histone H3 acetylation cooperates with mismatch repair and replicative polymerases in maintaining genome stability. PLoS Genet..

[38] Stejskal S., Stepka K., Tesarova L., Stejskal K., Matejkova M., Simara P., Zdrahal Z., Koutna I. (2015). Cell cycle-dependent changes in H3K56ac in human cells. Cell Cycle.

[39] Park Y.N., Morales D., Rubinson E.H., Masison D., Eisenberg E., Greene L.E. (2012). Differences in the curing of [PSI+] prion by various methods of Hsp104 inactivation. PLoS ONE.

[40] Ferreira P.C., Ness F., Edwards S.R., Cox B.S., Tuite M.F. (2001). The elimination of the yeast [PSI+] prion by guanidine hydrochloride is the result of Hsp104 inactivation. Mol. Microbiol..

[41] Romanova N.V., Chernoff Y.O. (2009). Hsp104 and prion propagation. Protein Pept. Lett..

[42] Ness F., Ferreira P., Cox B.S., Tuite M.F. (2002). Guanidine hydrochloride inhibits the generation of prion “seeds” but not prion protein aggregation in yeast. Mol. Cell Biol..

[43] Breslow D.K., Cameron D.M., Collins S.R., Schuldiner M., Stewart-Ornstein J., Newman H.W., Braun S., Madhani H.D., Krogan N.J., Weissman J.S. (2008). A comprehensive strategy enabling high-resolution functional analysis of the yeast genome. Nat. Methods.

[44] Shilatifard A. (2012). The COMPASS family of histone H3K4 methylases: Mechanisms of regulation in development and disease pathogenesis. Annu. Rev. Biochem..

[45] Schneider R., Bannister A.J., Myers F.A., Thorne A.W., Crane-Robinson C., Kouzarides T. (2004). Histone H3 lysine 4 methylation patterns in higher eukaryotic genes. Nat. Cell Biol..

[46] Nguyen A.T., Zhang Y. (2011). The diverse functions of Dot1 and H3K79 methylation. Genes Dev..

[47] Zhang T., Cooper S., Brockdorff N. (2015). The interplay of histone modifications—Writers that read. EMBO Rep..

[48] Vitrenko Y.A., Pavon M.E., Stone S.I., Liebman S.W. (2007). Propagation of the [PIN+] prion by fragments of Rnq1 fused to GFP. Curr. Genet..

[49] Wickner R.B., Dyda F., Tycko R. (2008). Amyloid of Rnq1p, the basis of the [PIN+] prion, has a parallel in-register beta-sheet structure. Proc. Natl. Acad. Sci. USA.

[50] Goehler H., Droge A., Lurz R., Schnoegl S., Chernoff Y.O., Wanker E.E. (2010). Pathogenic polyglutamine tracts are potent inducers of spontaneous Sup35 and Rnq1 amyloidogenesis. PLoS ONE.

[51] Sharma J., Liebman S.W. (2013). Exploring the basis of [PIN(+)] variant differences in [PSI(+)] induction. J. Mol. Biol..

[52] Topal S., Vasseur P., Radman-Livaja M., Peterson C.L. (2019). Distinct transcriptional roles for Histone H3-K56 acetylation during the cell cycle in Yeast. Nat. Commun..

[53] Dastidar R.G., Hooda J., Shah A., Cao T.M., Henke R.M., Zhang L. (2012). The nuclear localization of SWI/SNF proteins is subjected to oxygen regulation. Cell Biosci..

[54] Du Z., Park K.W., Yu H., Fan Q., Li L. (2008). Newly identified prion linked to the chromatin-remodeling factor Swi1 in Saccharomyces cerevisiae. Nat. Genet..

[55] Kryndushkin D., Wickner R.B., Shewmaker F. (2011). FUS/TLS forms cytoplasmic aggregates, inhibits cell growth and interacts with TDP-43 in a yeast model of amyotrophic lateral sclerosis. Protein Cell.

[56] Tibshirani M., Tradewell M.L., Mattina K.R., Minotti S., Yang W., Zhou H., Strong M.J., Hayward L.J., Durham H.D. (2015). Cytoplasmic sequestration of FUS/TLS associated with ALS alters histone marks through loss of nuclear protein arginine methyltransferase 1. Hum. Mol. Genet..

[57] Fukumoto T., Park P.H., Wu S., Fatkhutdinov N., Karakashev S., Nacarelli T., Kossenkov A.V., Speicher D.W., Jean S., Zhang L. (2018). Repurposing Pan-HDAC Inhibitors for ARID1A-Mutated Ovarian Cancer. Cell Rep..

[58] Rowbotham S.P., Barki L., Neves-Costa A., Santos F., Dean W., Hawkes N., Choudhary P., Will W.R., Webster J., Oxley D. (2011). Maintenance of silent chromatin through replication requires SWI/SNF-like chromatin remodeler SMARCAD1. Mol. Cell.

[59] Du H.N., Fingerman I.M., Briggs S.D. (2008). Histone H3 K36 methylation is mediated by a trans-histone methylation pathway involving an interaction between Set2 and histone H4. Genes Dev..

[60] Ng H.H., Xu R.M., Zhang Y., Struhl K. (2002). Ubiquitination of histone H2B by Rad6 is required for efficient Dot1-mediated methylation of histone H3 lysine 79. J. Biol. Chem..

[61] Sun Z.W., Allis C.D. (2002). Ubiquitination of histone H2B regulates H3 methylation and gene silencing in yeast. Nature.

[62] Malovichko Y.V., Antonets K.S., Maslova A.R., Andreeva E.A., Inge-Vechtomov S.G., Nizhnikov A.A. (2019). RNA Sequencing Reveals Specific TranscriptomicSignatures Distinguishing Effects of the [SWI(+)] Prion and SWI1 Deletion in Yeast Saccharomyces cerevisiae. Genes.

[63] Treusch S., Lindquist S. (2012). An intrinsically disordered yeast prion arrests the cell cycle by sequestering a spindle pole body component. J. Cell Biol..

[64] Liebman S.W., Chernoff Y.O. (2012). Prions in yeast. Genetics.

[65] Du Z., Goncharoff D.K., Cheng X., Li L. (2017). Analysis of [SWI(+) ] formation and propagation events. Mol. Microbiol..

[66] Tanaka M., Chien P., Naber N., Cooke R., Weissman J.S. (2004). Conformational variations in an infectious protein determine prion strain differences. Nature.

[67] Bennett S.A., Cobos S.N., Meykler M., Fallah M., Rana N., Chen K., Torrente M.P. (2019). Characterizing Histone Post-translational Modification Alterations in Yeast Neurodegenerative Proteinopathy Models. J. Vis. Exp..

[68] Alberti S., Halfmann R., Lindquist S. (2010). Biochemical, cell biological, and genetic assays to analyze amyloid and prion aggregation in yeast. Methods Enzymol..

[69] Kobayashi O., Suda H., Ohtani T., Sone H. (1996). Molecular cloning and analysis of the dominant flocculation gene FLO8 from Saccharomyces cerevisiae. Mol. Gen. Genet..

[70] Derkatch I.L., Bradley M.E., Zhou P., Chernoff Y.O., Liebman S.W. (1997). Genetic and environmental factors affecting the de novo appearance of the [PSI+] prion in Saccharomyces cerevisiae. Genetics.

[71] Winzeler E.A., Shoemaker D.D., Astromoff A., Liang H., Anderson K., Andre B., Bangham R., Benito R., Boeke J.D., Bussey H. (1999). Functional characterization of the S. cerevisiae genome by gene deletion and parallel analysis. Science.

