# Supplementary material for: [PRION+] States Are Associated with Specific Histone H3 Post-Translational Modification Changes
Source: Pathogens. 2022 Nov 29;11(12):1436. doi: 10.3390/pathogens11121436 (PMC9786042; doi:10.3390/pathogens11121436)
Supplement: Supplementary file 1 [file pathogens-11-01436-s001.zip › pathogens-1910818-SI.pdf]

Supplemental Materials.

Table S1. List of yeast strains used in this study.

Strain	Genotype	Reference
[swi$^-$]	*MATα his3Δ1 leu2Δ0 met15Δ0 ura3Δ0 flo8::FLO8::HIS3 [swi$^-$]*	[64]
[SWI$^+$]	*MATα his3Δ1 leu2Δ0 met15Δ0 ura3Δ0 flo8::FLO8::HIS3 [SWI$^+$]*	[64]
Swi1 DAmP	*MATα his3Δ1 leu2Δ0 ura3Δ0 met15Δ0 cyh2*	[43]
BY4741 [pin$^-$]	*MAT*a *his3-Δ1 leu2-Δ met15-Δ ura3-Δ [psi−][pin$^-$]*	[70]
BY4741 [PIN$^+$]	*MAT*a *his3-Δ1 leu2-Δ met15-Δ ura3-Δ [psi$^-$][PIN$^+$]*	[70]
ΔRnq1	*MATα his3Δ1 leu2Δ0 ura3Δ0 met15Δ0*	[71]
74D [pin$^-$]	*MATα*, *ade1-14*(UGA), *his3*, *leu2*, *trp1*, *ura3 [pin$^-$]*	[66]
74D [PIN$^+$]	*MATα*, *ade1-14*(UGA), *his3*, *leu2*, *trp1*, *ura3 [PIN$^+$]*	[66]
BY4741	*MATα his3Δ1 leu2Δ0 ura3Δ0 met15Δ0*	[71]
ΔGcn5	*MATα his3Δ1 leu2Δ0 ura3Δ0 met15Δ0*	[71]
ΔRtt109	*MATα his3Δ1 leu2Δ0 ura3Δ0 met15Δ0*	[71]
ΔSet2	*MATα his3Δ1 leu2Δ0 ura3Δ0 met15Δ0*	[71]

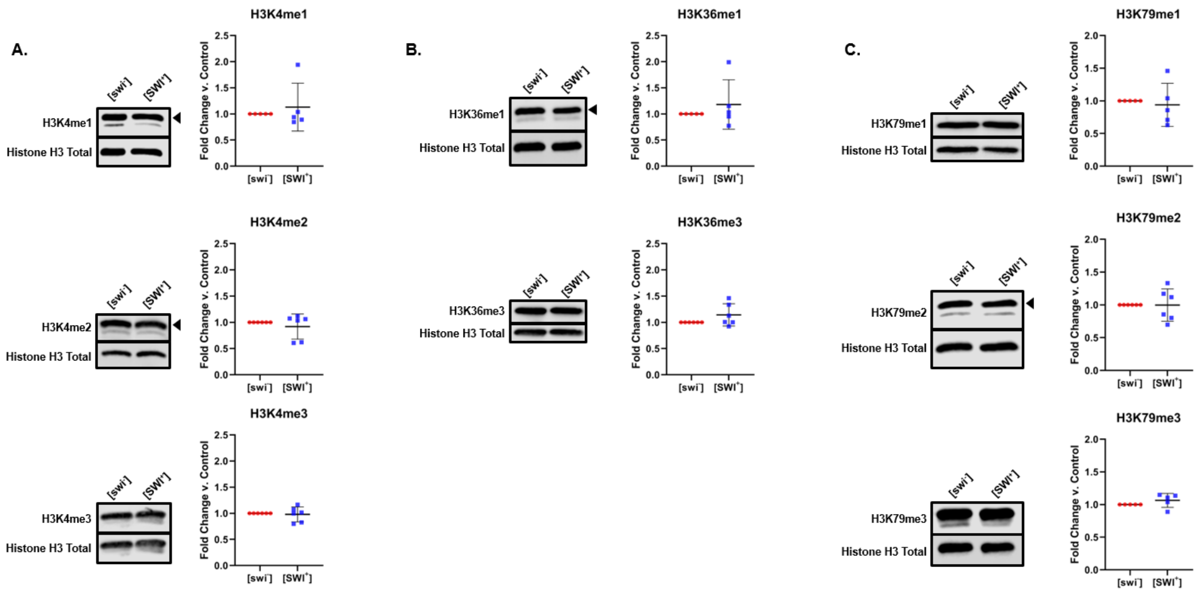

Figure S1. Methylation levels on lysines 4, 36, and 79 of histone H3 are stable in connection to [SWI$^+$]. Representative blots probing for (A) H3K4me1/me2/me3, (B) H3K36me1/me3, and (C) H3K79me1/me2/me3 levels in [swi$^-$] and [SWI$^+$] yeast. Graphs compiling quantification for multiple biological replicates are shown alongside blots. Graphs show mean fold changes as well as individual data points. Error bars represent ±SD. (n=5-6)

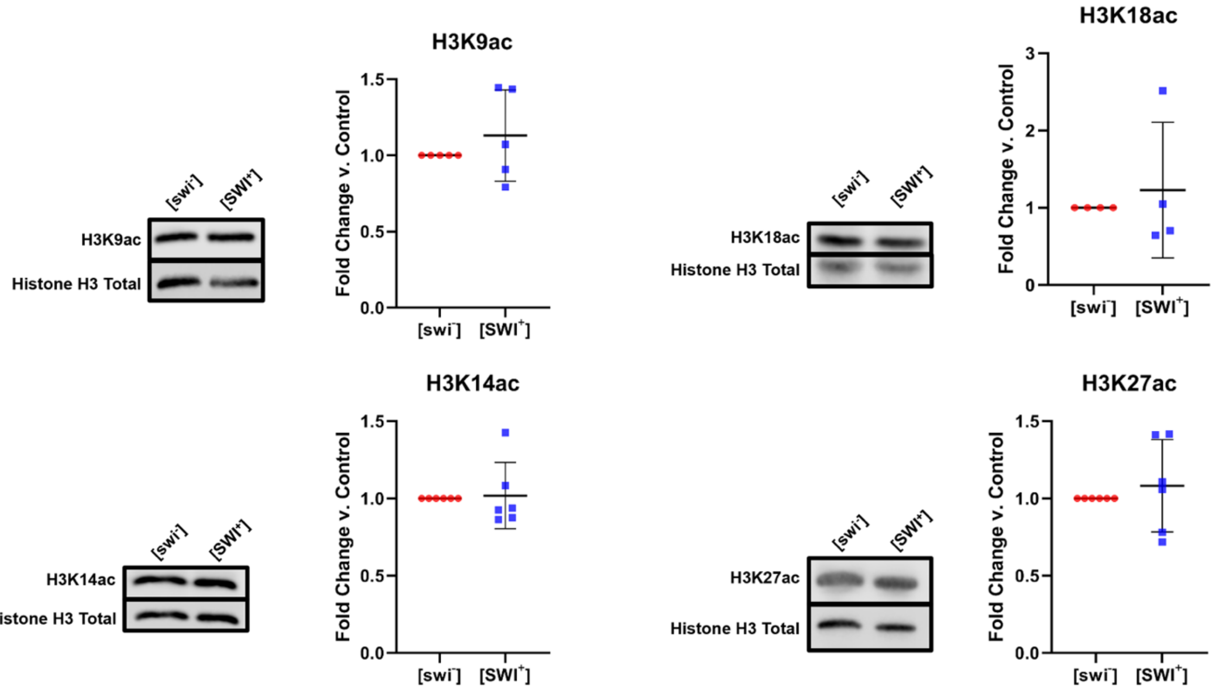

Figure S2. Levels of acetylation on lysines 9, 18, 14, and 27 of Histone H3 are unchanged in connection to [SWI⁺]. Representative blots probing for H3K9ac, H3K14ac, H3K18ac, and H3K27ac levels in [swi⁻] and [SWI⁺] yeast. Graphs compiling quantification for multiple biological replicates are shown alongside blots. Graphs show mean fold changes as well as individual data points. Error bars represent ±SD. (n=4-6)

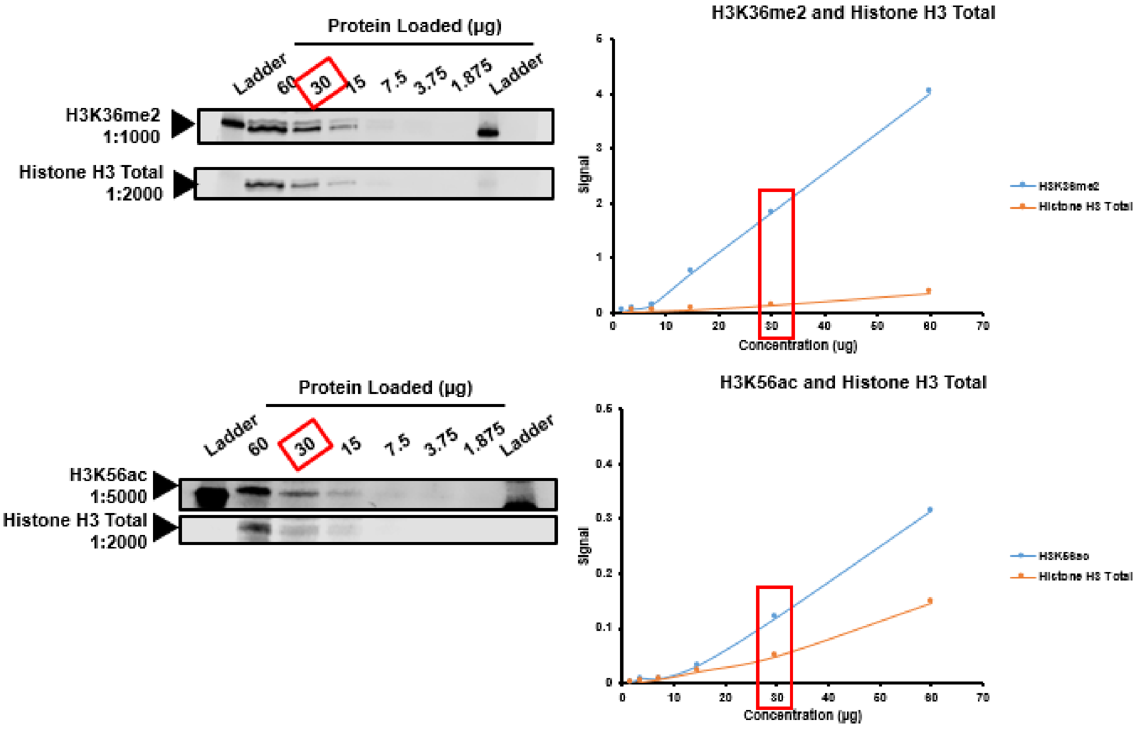

Figure S3. Linear range of detection for select antibodies. Varying protein amounts were assayed with α-H3K36me2, and α-H3K56ac antibodies. α-Histone H3 Total was used as a loading control. Graphs show amount of protein loaded (µg) vs. signal intensity. Red boxes indicate amount of protein loaded in all experiments.

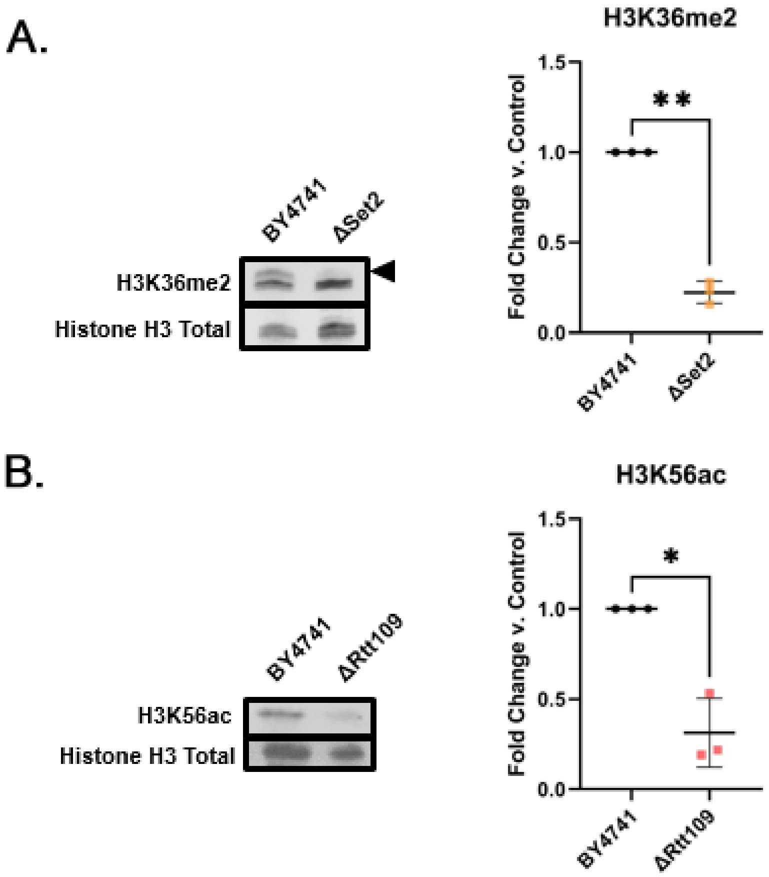

Figure S4. Deletion of histone modifying enzymes leads to the loss of specific histone post-translational modifications. Antibody specificity for selected modifications was assayed through deletion strains of individual histone modifying enzymes. Representative blots show (A) ΔSet2 yeast compared to BY4741 wild-type yeast probing for H3K36me2, and (B) ΔRtt109 yeast compared to BY4741 wild-type yeast probing for H3K56ac. Graph showing mean fold changes as well as individual data points for multiple biological replicates accompany each blot. Error bars represent ±SD. (n=3)

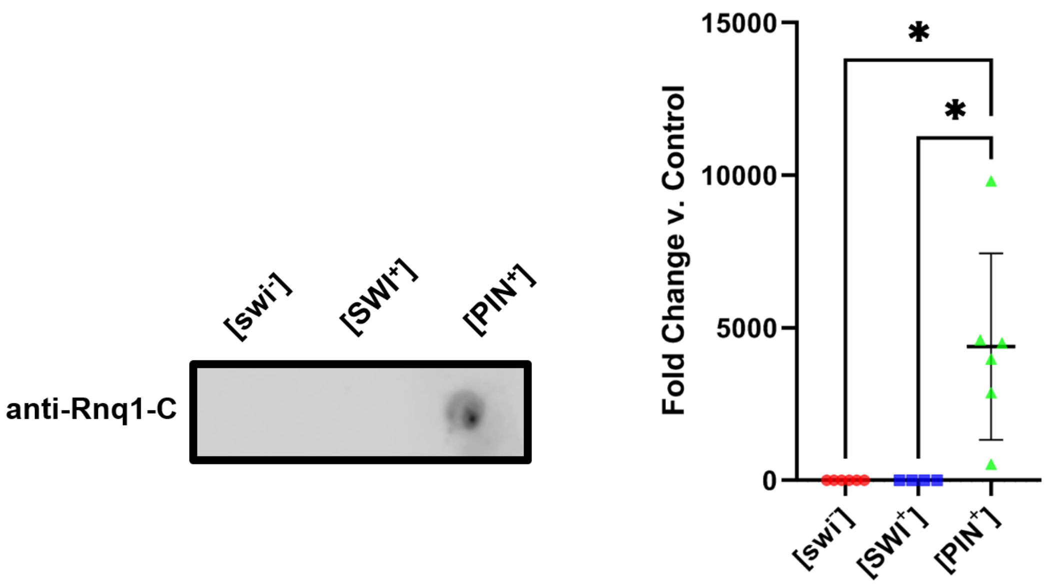

Figure S5. [SWI+] yeast do not display Rnq1 aggregates. Filter retention assays against Rnq1 aggregates were performed in [swi-] and [SWI+] yeast. [PIN+] yeast were used as a positive control. Quantifications indicate relative density normalized to [swi-] signal. Error bars represent ±SD. (n=6) * = $p<0.05$.

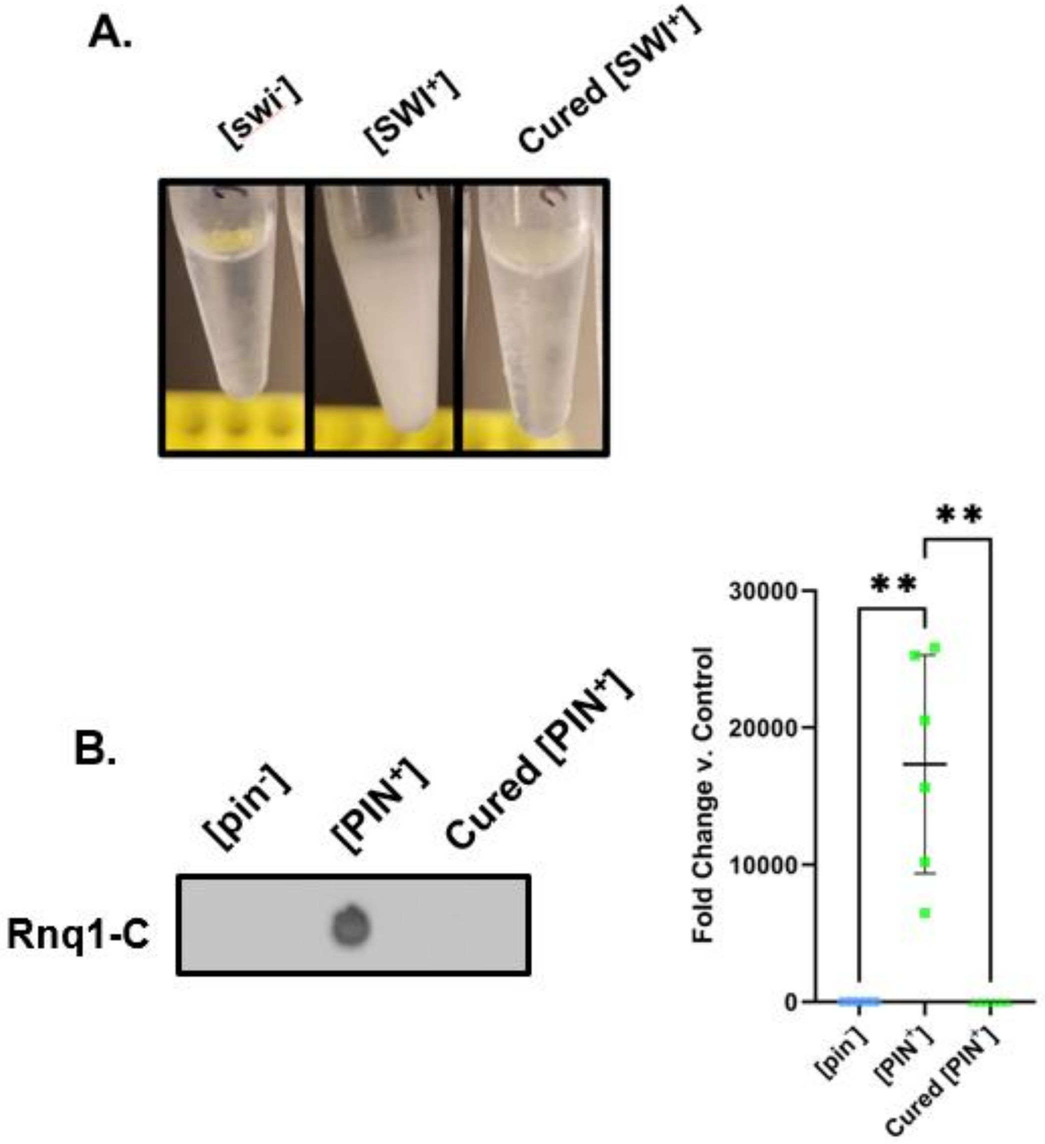

Figure S6. Treatment of [PRION$^+$] yeast with guanidine hydrochloride abolishes prion phenotypes. (A) [swi$^-$], [SWI$^+$] and [SWI$^+$] yeast treated with 1mM GuHCl after resuspension in synthetic dropout media lacking histidine. (B) Filter retention assay probing for Rnq1 aggregation in [pin$^-$] and [PIN$^+$] yeast, as well as [PIN$^+$] treated with 1mM GuHCl. Quantifications indicate relative density normalized to [swi$^-$] signal. Error bars represent ±SD. n=6 for each experiment.

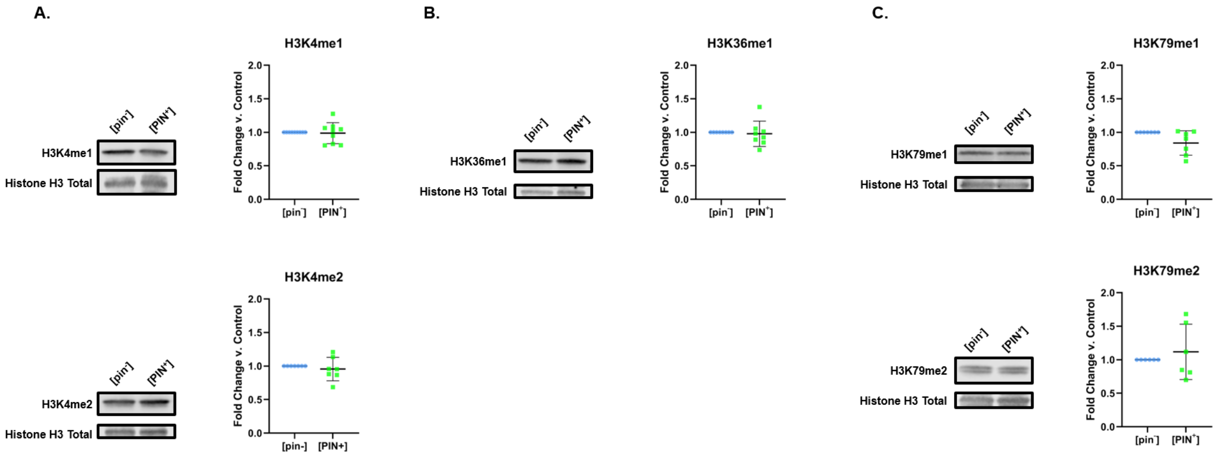

Figure S7. Levels of mono- and dimethylation of lysines 4 and 79, as well as mono-methylation on lysine 36 on histone H3 remain unchanged in the context of [PIN$^+$]. Representative blots probing for (A) H3K4me1/me2, (B) H3K36me1, and (C) H3K79me1/me2 levels in [pin$^-$] and [PIN$^+$] yeast. Graphs compiling quantification of multiple biological replicates are shown alongside blots. Graphs show mean fold changes as well as individual data points. Error bars represent ±SD. (n=6-9)

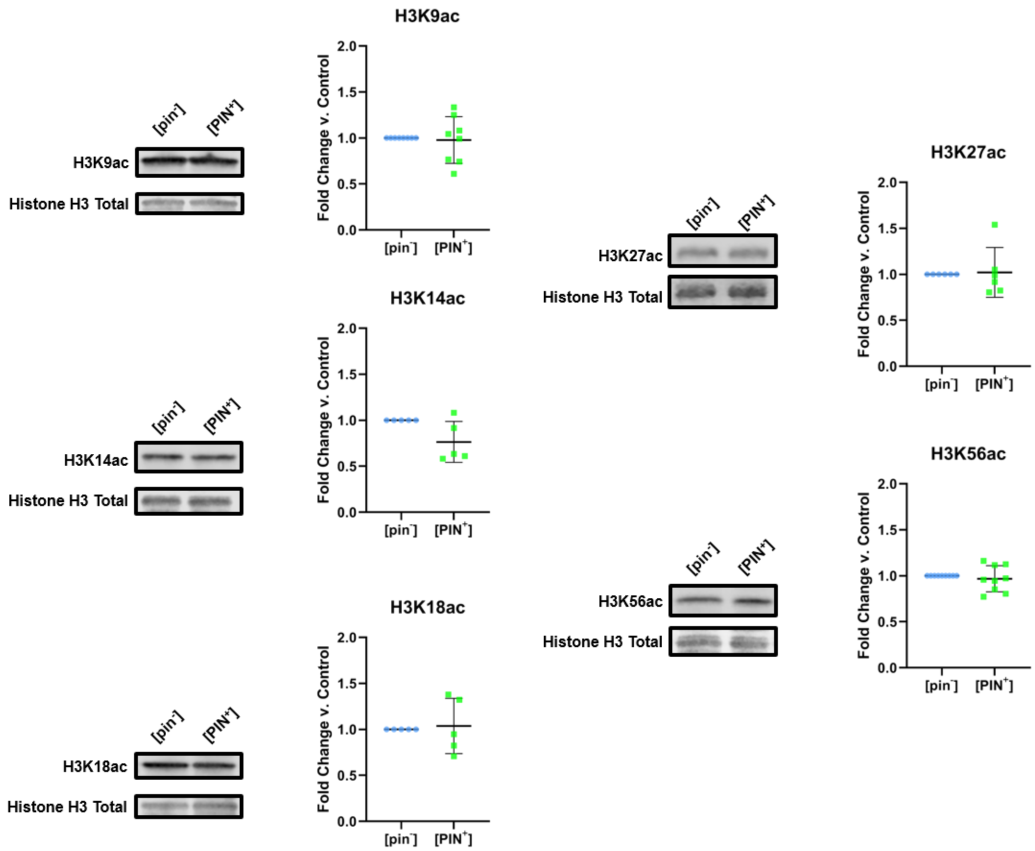

Figure S8. Levels of acetylation of lysines 9, 14, 18, 27 and 56 on histone H3 remain unchanged in the context of [PIN$^+$] yeast. Representative blots probing for changes in H3K9ac, H3K14ac, H3K18ac, H3K27ac, and H3K56ac levels in [pin$^-$] and [PIN$^+$] yeast. Graphs compiling quantification of multiple biological replicates are shown alongside blots. Graphs show mean fold changes as well as individual data points. Error bars represent ±SD. (n=5-10)

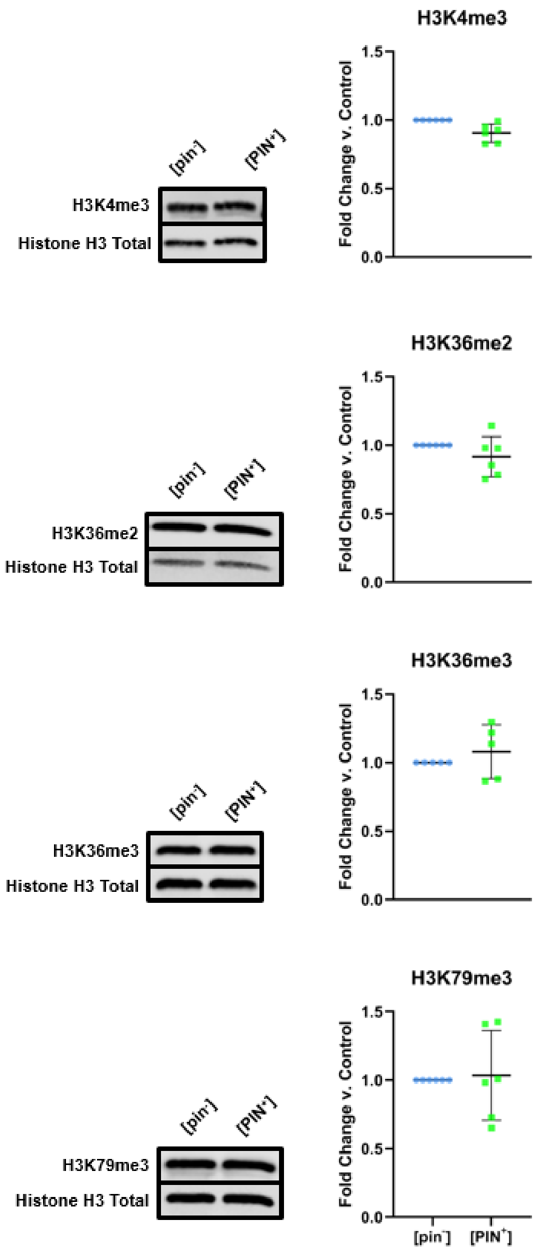

Figure S9. Histone post-translational modification landscape changes caused by [PRION+] diverge in different genetic backgrounds. 74D-694 yeast bearing either [pin-] or [PIN+] were probed for changes in H3K4me3, H3K36me2, H3K36me3, and H3K79me3 levels through immunoblotting. Graphs compiling quantification of multiple biological replicates are shown alongside blots. Graphs show showing mean fold changes as well as individual data points. Error bars represent ±SD. (n=5-6)

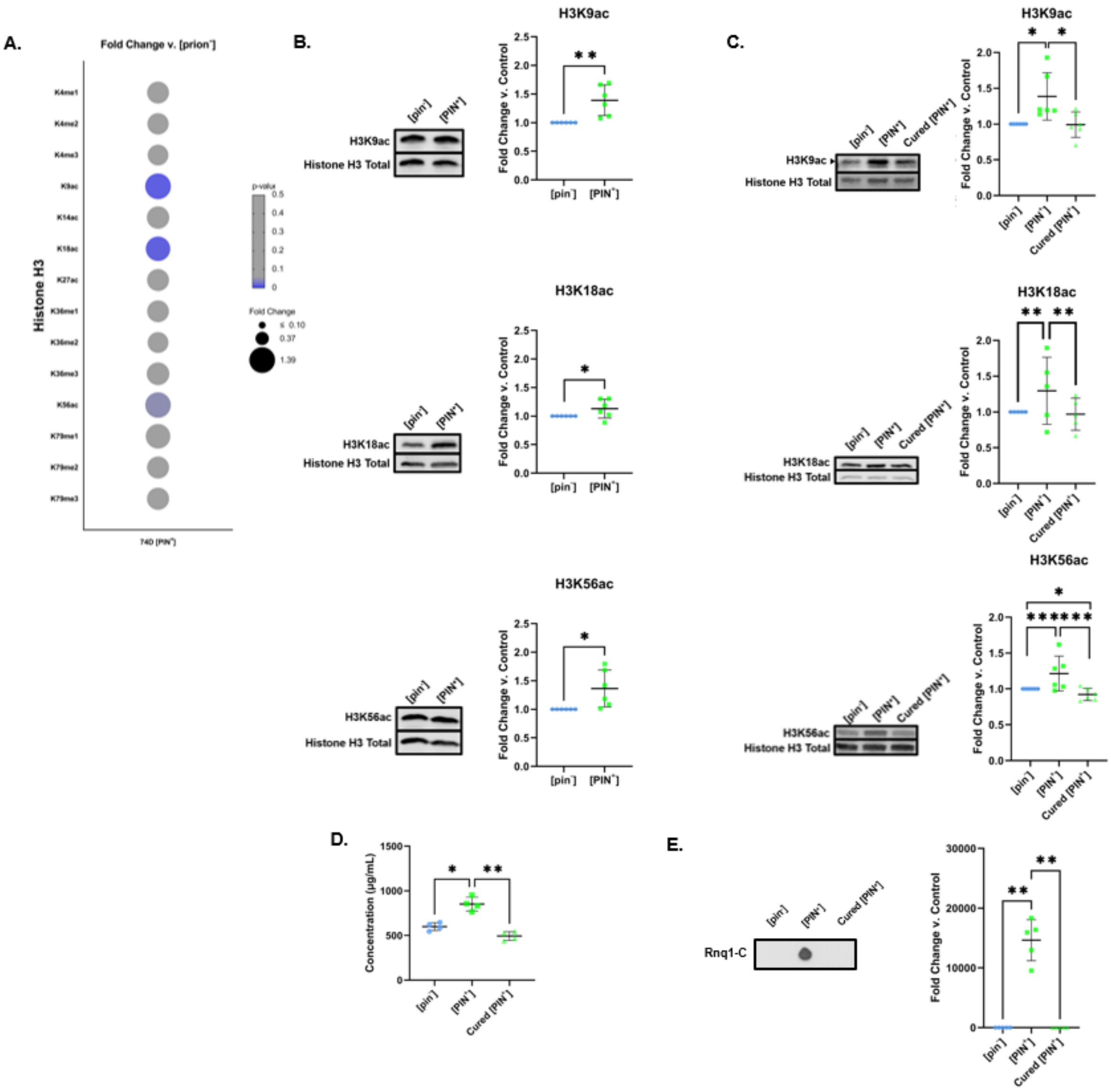

Figure S10. Histone post-translational modification landscape changes linked to [PIN$^+$] diverge in different genetic backgrounds. (A) Bubble plot showing overall histone H3 post-translational modification level fold changes in 74D-694 [PIN$^+$] yeast compared to [pin$^-$] controls. Size of bubbles represents relative fold changes in histone PTM levels. *p* values were calculated using a two-tailed *t* test with Welch's modification. Color scale represents *p* values; gray indicates a $p > 0.05$, while blue indicates a statistically significant increase with a *p* value $\leq$ 0.05. (B) Representative immunoblots reveal significant increases in the levels of H3K9ac, H3K18ac, and H3K56ac levels in 74D-694 [PIN$^+$] yeast. (C) Representative immunoblots showing the levels of H3K9ac, H3K18ac, and H3K56ac in 74D-694 [pin$^-$], [PIN$^+$], and cured [PIN$^+$] yeast are shown. Treatment with 1mM GuHCl restored histone PTM levels to those seen in [pin$^-$] yeast. Graphs compiling quantification of multiple biological replicates are shown alongside blots. Graphs show showing mean fold changes as well as individual data points. (D) Total RNA was quantified in 74D-694 [pin$^-$], [PIN$^+$], and cured [PIN$^+$] yeast using a Qubit

Fluorometer. Quantifications indicate raw concentrations obtained from Qubit in µg/mL. (E) Representative filter assay probing 74D-694 [pin$^-$], [PIN$^+$], and cured [PIN$^+$] yeast for Rnq1 aggregates. Quantifications indicate relative density normalized to [swi$^-$] signal. Quantification compiling multiple biological replicates is also shown. Error bars for all graphs represent ±SD. (n = 3-7) * = p<0.05, ** = p<0.01, *** = p<0.001.

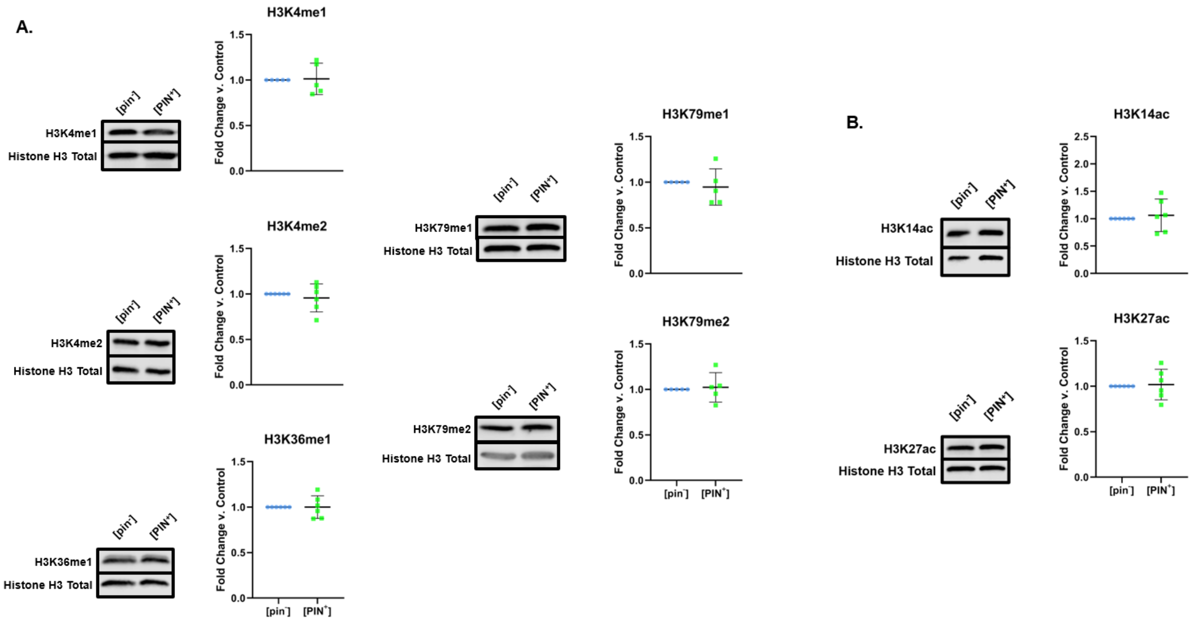

Figure S11. [PIN$^+$] does not impact select methylation and acetylation levels of histone H3 in 74D-694 yeast. Representative blots for (A) H3K4me1/me2, H3K36me1, and H3K79me1/me2, and (B) H3K14ac and H3K27ac levels in 74D-694 [pin$^-$] and [PIN$^+$] yeast are shown. Graphs compiling quantification of multiple biological replicates are shown alongside blots. Graphs show showing mean fold changes as well as individual data points. Error bars represent ±SD. (n=4-6)

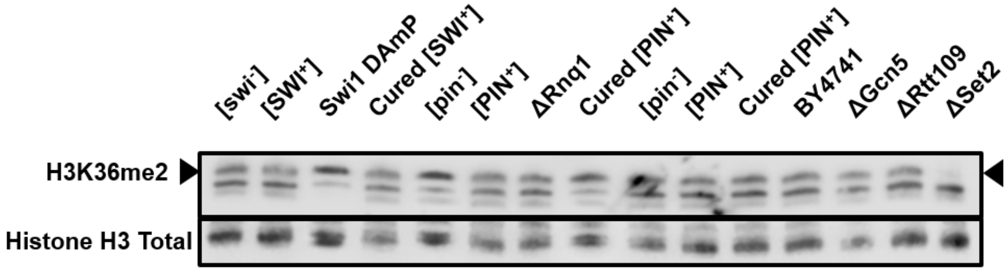

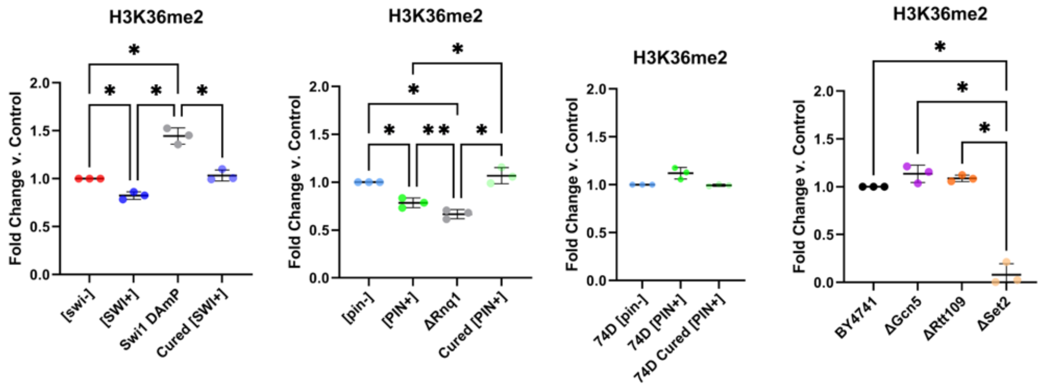

Figure S12. Changes in H3K36me2 in various yeast strains. Representative blot probing for changes in levels of H3K36me2 levels in [swi$^-$], [SWI$^+$], Swi DAmP, cured [SWI$^+$], [pin$^-$], [PIN$^+$], ΔRnq1, cured [PIN$^+$], 74D-694 [pin$^-$], 74D-694 [PIN$^+$] and 74D-694 cured [PIN$^+$] strains, as well as strains harboring deletions of the histone modifying enzymes Gcn5, Rtt109, and Set2. A ΔSet2 deletion strain highlights H3K36me2 blot band. Graphs compiling quantification of multiple biological replicates are shown alongside blots. Graphs show showing mean fold changes as well as individual data points. Error bars represent ±SD. (n=3)

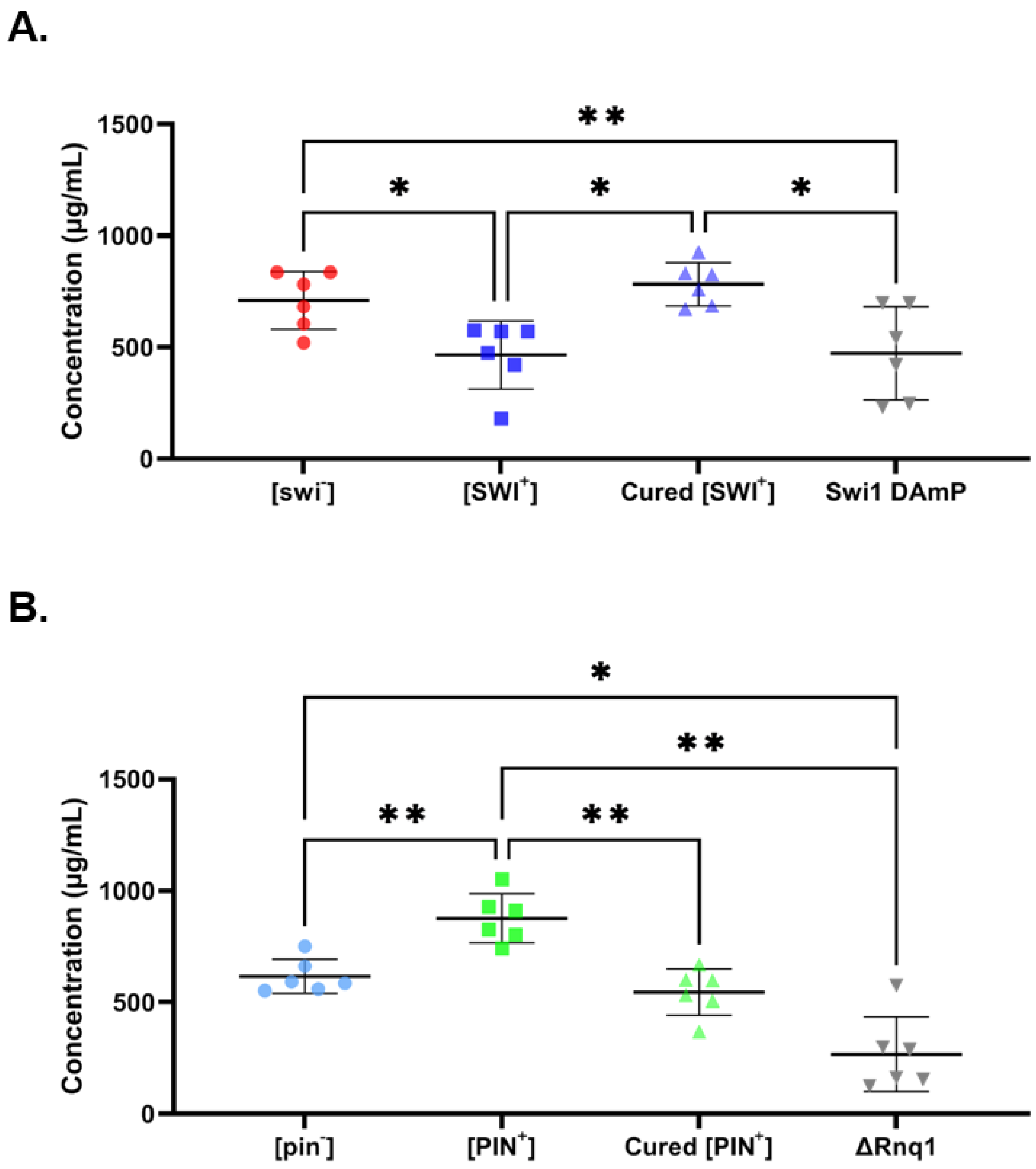

Figure S13. Total RNA levels in [PRION+] yeast differ from those in [prion-] yeast. Total RNA levels in (A)[swi-], [SWI+], cured [SWI+] and Swi1 DAmP yeast, or (B) [pin-], [PIN+], cured [PIN+] and ΔRnq1 yeast. Quantifications indicate raw concentrations obtained from Qubit in µg/mL. Error bars represent ± SD. n = 6, * = p<0.05; ** = p<0.01.

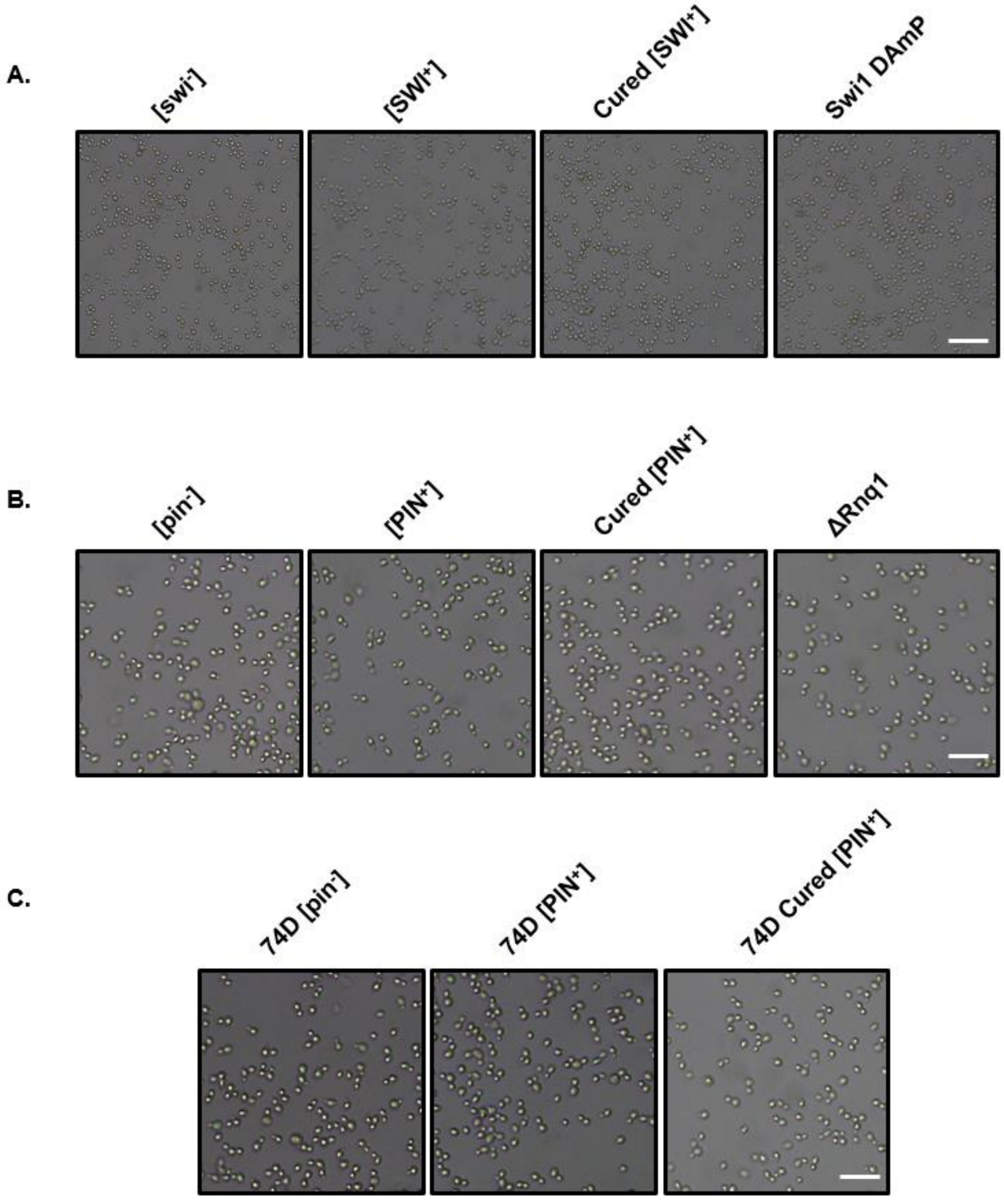

Figure S14. Cell morphology is similar in [prion$^-$], [PRION$^+$], cured [PRION$^+$], and loss-of-function yeast. Zymolyase-treated (A) [Swi1$^-$], [SWI$^+$], cured [SWI$^+$] and Swi1 DAmP, (B) [pin$^-$], [PIN$^+$], cured [PIN$^+$] and Rnq1 or (C) 74D-694 [pin$^-$], 74D-694 [PIN$^+$] and cured 74D-694 [PIN$^+$] cells were imaged at 5x magnification. Scale bars represent 20μm.
